# Geographic and sociodemographic access to systemic anticancer therapies for secondary breast cancer: a systematic review

**DOI:** 10.1186/s13643-023-02382-3

**Published:** 2024-01-18

**Authors:** Sally Anne Pearson, Sally Taylor, Antonia Marsden, Jessica Dalton O’Reilly, Ashma Krishan, Sacha Howell, Janelle Yorke

**Affiliations:** 1 Division of Nursing, Midwifery and Social Work, The Christie NHS Foundation Trust, University of Manchester, Oxford Rd, Manchester, M13 9PL UK; 2 Christie Patient Centred Research, The Christie NHS Foundation Trust, 550 Wilmslow Road, Manchester, M20 4BX UK; 3 Division of Population Health, Health Services Research and Primary Care, University of Manchester, Oxford Rd, Manchester, M13 9PL UK; 4 Division of Cancer Sciences, University of Manchester, Oxford Rd, Manchester, M13 9PL UK; 5 Division of Nursing, Midwifery and Social Work, University of Manchester, Oxford Rd, Manchester, M13 9PL UK

## Abstract

**Background:**

The review aimed to investigate geographic and sociodemographic factors associated with receipt of systemic anticancer therapies (SACT) for women with secondary (metastatic) breast cancer (SBC).

**Methods:**

Included studies reported geographic and sociodemographic factors associated with receipt of treatment with SACT for women > 18 years with an SBC diagnosis. Information sources searched were Ovid CINAHL, Ovid MEDLINE, Ovid Embase and Ovid PsychINFO. Assessment of methodological quality was undertaken using the Joanna Briggs Institute method. Findings were synthesised using a narrative synthesis approach.

**Results:**

Nineteen studies published between 2009 and 2023 were included in the review. Overall methodological quality was assessed as low to moderate. Outcomes were reported for treatment receipt and time to treatment. Overall treatment receipt ranged from 4% for immunotherapy treatment in one study to 83% for systemic anticancer therapies (unspecified). Time to treatment ranged from median 54 days to 95 days with 81% of patients who received treatment < 60 days.

Younger women, women of White origin, and those women with a higher socioeconomic status had an increased likelihood of timely treatment receipt. Treatment receipt varied by geographical region, and place of care was associated with variation in timely receipt of treatment with women treated at teaching, research and private institutions being more likely to receive treatment in a timely manner.

**Conclusions:**

Treatment receipt varied depending upon type of SACT. A number of factors were associated with treatment receipt. Barriers included older age, non-White race, lower socioeconomic status, significant comorbidities, hospital setting and geographical location. Findings should however be interpreted with caution given the limitations in overall methodological quality of included studies and significant heterogeneity in measures of exposure and outcome. Generalisability was limited due to included study populations.

Findings have practical implications for the development and piloting of targeted interventions to address specific barriers in a socioculturally sensitive manner. Addressing geographical variation and place of care may require intervention at a commissioning policy level. Further qualitative research is required to understand the experience and of women and clinicians.

**Systematic review registration:**

PROSPERO CRD42020196490

**Supplementary Information:**

The online version contains supplementary material available at 10.1186/s13643-023-02382-3.

## Introduction

Over half a million deaths from metastatic or secondary breast cancer (SBC) were estimated worldwide in 2015 with an estimated prevalent population of 57,215 patients with SBC in England in 2021 [1, 2]. Secondary breast cancer is treatable however remains incurable, and despite recent treatment advances, median survival rates have remained stable at 2–3 years over recent years with a 5-year survival rate of 25% [3]. Secondary breast cancer (SBC) has been defined as the development of new tumours in tissues and organs away from the primary tumour site. The most common sites of metastases are lungs, the liver, bones and the brain [4]. Treatment for SBC aims to improve overall survival, increase disease-free progression and improve quality of life whilst balancing toxicities associated with treatment [5]. The majority of patients with SBC receive SACT which may be chemotherapy, targeted therapy, immunotherapy and/or endocrine therapy. Treatment selection is most often guided by tumour biology, and clinical factors and clinical decisions regarding SACT are usually influenced by hormone expression and human epidermal growth factor receptor 2 (HER2) status combined with patient preference, prior therapy (and tolerability), comorbidities and organ function [6]. Receipt of appropriate treatment which is concordant with current, evidence-based clinical guidelines has been associated with improved clinical outcomes for patients with SBC [7, 8]. Treatment disparities related to geographical location have been reported which if left unaddressed may lead to unwarranted variation in outcomes and suggest that geographical access to cancer services remains a concern [9, 10]. The nature and extent of these geographical disparities for women with SBC remain poorly understood.

Addressing geographical disparity is synonymous with promoting equitable access to cancer treatment. At its most, fundamental access to health care has been described as entry into or use of the health care system characterised by those factors which influence entry or use. These include physician numbers and capacity, spatial and geographic relationships between providers of health and the ease with which people can use care, which included clinic hours, waiting time and length of waiting time for an appointment [11]. Contemporary theory of access has proposed a patient-centred framework which suggested that appropriate access to care and treatment occurs at the interface between individual, household and community factors and those of health care systems, institutions, organisations and providers. This was conceptualised in terms of dimensions of accessibility which included approachability, acceptability, availability and accommodation. Corresponding abilities of patients were identified which included a perceived need for health care, ability to seek and reach appropriate health care and the ability to pay combined with the ability to engage. It was proposed that these dimensions of access and abilities of persons interact to generate appropriate, equitable access [12]. The framework has been widely used and was selected as the theoretical framework to guide the review as it provided a contemporary approach to understanding and contextualising access which incorporates principles of patient-centred care of respectful, informed and appropriate care [13, 14]. The framework has been widely cited and was developed through a synthesis of the published literature [15]. The framework provided a useful conceptualisation for classifying individual factors and clinical characteristics and their interface with wider contextual factors to develop a greater understanding of geographic and sociodemographic access to SACT for the treatment of SBC.

A comprehensive understanding of factors associated with equitable access to SACT for women with SBC is lacking, and to date, there has been no systematic review of the evidence. A systematic review was deemed an appropriate approach to identify gaps in the current evidence and answer the clinical question related to geographic and socioeconomic factors associated with receipt of SACT for the treatment of SBC. The review sought to identify the international evidence to confirm current practice, identify any variation in access to SACT for the treatment of SBC and to identify and inform areas for future research. The review aimed to identify and examine individual, clinical and contextual factors with the intent to measure association between sociodemographic, clinical and geographic factors and receipt of systemic anticancer therapy (SACT) for the treatment of secondary breast cancer (SBC) identifying factors which may act as barriers and enabling factors for receipt of guideline concordant SACT.

### Objectives

- To review the available evidence to investigate factors associated with receipt of SACT for the treatment of SBC
- Identify barriers and enabling factors for treatment access and receipt of SACT.
- Explore women and clinicians experience of access and treatment receipt for secondary breast cancer.

## Methods

The published review protocol [16] was developed in accordance with the Joanna Briggs Institute guidance (JBI) for developing a mixed-methods systematic review protocol [17]. The protocol was reported in accordance with the Preferred Reporting Items for Systematic Reviews and Meta-Analyses for systematic review protocols (PRISMA-P) extension statement [18].

No qualitative studies were identified for inclusion. On this basis, it was not possible to address the objective to explore women and clinicians experience of access and treatment receipt for SBC. This necessitated amendment to the published protocol for assessment of methodological quality and risk of bias and data analysis and synthesis. The method reported here is the amended version of the protocol which was conducted and reported in accordance with the Preferred Reporting Items for Systematic Reviews and Meta-Analyses (PRISMA) statement [19].

### Eligibility criteria

Study eligibility was predefined using a modified version of the population, intervention, comparison and outcomes (PICO) framework [20]. Population was women > 18 with an SBC diagnosis. Intervention/exposures were individual factors related to age, gender, sexual orientation, race/ethnicity, socioeconomic status, education, language and literacy and psychosocial characteristics. Clinical characteristics included clinical subtype of disease which included hormone receptor status, HER2 status and previous treatment response. Contextual factors included geographical location, distance, travel time and health care system factors. Comparators were those of standard of care, and the outcome was defined as receipt/nonreceipt of SACT. Studies were ineligible for inclusion where they reported on women with primary/early stage breast cancer only. Males with a secondary breast cancer diagnosis were ineligible as this was classified as a rare disease beyond the scope of the review. Studies which reported comparative treatment effect and efficacy were ineligible as the primary outcome of interest was access, receipt and utilisation of SACT (Additional file 1).

### Information sources

Preliminary searches were undertaken in May 2020, updated 20 June 2022 and the final searches reported in the current paper were undertaken on 24 August 2023. Electronic databases searched were Ovid CINAHL, Ovid MEDLINE, Ovid Embase and Ovid PsychINFO accessed through the University of Manchester for original searches and The Christie NHS Foundation Trust library for final searches. NHS Evidence was searched for unpublished studies and grey literature, and the Joanna Briggs Institute Evidence-Based Practice database (JBI EBP) and the Cochrane Library were searched in the original searches. The Cochrane Library was searched in the final searches. Reference lists of included studies were searched. Searches were developed and undertaken with the support of a medical librarian/evidence specialist.

### Search strategy

The updated search strategy was developed in accordance with guidance set out in the JBI Manual for Evidence Synthesis. This included the identification of key words in a preliminary (unpublished) nonsystematic review of the literature and analysis of text words contained in the titles and abstracts of papers and of the index terms used in a bibliographic database to describe relevant articles [21]. This informed the development of the search strategy which was conceptually structured using the modified population, intervention, comparison and outcomes (PICO) framework [20]. A validated filter was used for geographic and sociodemographic factors [22]. This filter was applied to updated searches. Searches were limited to English language and studies published from 2000 onwards to include contemporary studies which reflected current trends in access to SACT for SBC. Final updated searches are reported in accordance with the PRISMA-S extension to the PRISMA statement for reporting literature searches in systematic reviews [19, 23] (Additional files 2 and 3). Final updated searches were conducted on 24 August 2023 and were revised and updated to reflect peer reviewer comments.

### Study selection

Study titles and abstracts were reviewed against the predefined inclusion and exclusion criteria by the lead author and second reviewer (S. P., J. D. O.). Any disagreements were resolved through discussion. Records were stored and managed in EndNote X9. Potentially eligible studies were retrieved and full text assessed by the two reviewers. Final study selection was approved by the supervisory panel (S. T., A. M., J. Y.). Studies which did not meet inclusion criteria were excluded with reasons for exclusion recorded.

### Data collection

Data were extracted and recorded in MS Excel using an adaptation of a standardised data extraction tool which was piloted prior to use. Data extraction was undertaken by the author (S. P.) and second reviewer (J. D. O.). Data for author, year of publication, study design, setting, country, primary data source and study population were extracted. Baseline population demographics for age, race, ethnicity and socioeconomic status were extracted along with primary exposure variables and covariates. Clinical characteristics for diagnosis, clinical subtype and comorbidities were extracted, and contextual factors for place of care, geographical location and population density were extracted, where reported. Numbers for the proportion of women with a secondary breast cancer (SBC) diagnosis included in overall samples was also extracted. This was where the sample was not exclusively women with secondary disease. Data related to the outcome of relevance was extracted as a proportion of studies also reported other nonrelevant outcomes. This included the type of outcome measure, i.e. dichotomous (binary) or continuous, time to event data or a combination of these. Data were extracted from relevant statistical analysis including odds ratios (OR) and hazard ratios (HR) with corresponding confidence intervals (CI) and *P*-values where these were reported. Three study first authors were contacted to obtain additional data. One reply was received stating that the author no longer had access to additional study data.

### Assessment of methodological quality and risk of bias

Methodological quality was assessed using the relevant JBI critical appraisal checklists for cohort and analytical cross-sectional study designs respectively. This included an appraisal of the study population selection method, measures of outcome and relevant exposures, identification and management of potential confounding variables, validity and reliability of outcome measurement and follow-up strategies [24, 25]. The assessment of methodological quality was undertaken independently by the author and second reviewer (S. P., J. D. O.), and any disagreements were resolved through discussion. An overall GRADE quality rating [26] was applied on an individual study level based on the assessment of methodological quality and reporting clarity, specifically the proportion of SBC patients for whom data was reported separately and could be extracted. A weighting was applied to each study based on the overall assessment of methodological quality and the GRADE rating. This was taken into consideration in the analysis and synthesis and reflected in the ‘Discussion’ and ‘Conclusion’. Studies were not excluded based upon low methodological quality.

### Synthesis

Synthesis of study findings was undertaken using a narrative synthesis approach [27, 28]. Studies were descriptively summarised in terms of methodological quality, study characteristics and main finding relative to the review objectives. A systematic approach to the synthesis was taken to identify patterns of effects and similarities and differences between studies to provide a comprehensive account of included studies in narrative form. The narrative synthesis was structured around multi-level factors which have previously demonstrated association with access and timely receipt of treatment as the primary outcome measure for the review. This was determined a priori guided by the theoretical model of access adopted to guide the review [12]. Relationships within and between studies were analysed to explore similarities and differences based on different population groups, study settings, exposures, any variability in outcomes and study design. The published protocol specified that where possible, data would be pooled using statistical meta-analysis in accordance with guidance for meta-analysis of observational studies. Due to high levels of heterogeneity in measures of outcome and exposure within and between studies, meta-analysis was precluded.

## Results

### Study identification and selection

The final updated search undertaken on 24 August 2023 which updated previous searches from December 2020 to June 2022 identified 1693 titles and abstracts. Following removal of 195 duplicates, 1498 title and abstracts were screened against the inclusion and exclusion criteria. One-thousand three-hundred and eighty-five (1385) were excluded. One-hundred and thirteen (113) reports were sought for retrieval for full-text screening. Thirty-three reports were unavailable. Eighty (80) full-text articles were assessed for eligibility. Following full-text screening, 76 were excluded. Studies were excluded where it was not possible to identify secondary breast cancer patients in the analysis, where the primary outcome was not reported for women with SBC, where factors associated with access and receipt of treatment were not reported and where full-text or English language reports were not available. The remaining 4 studies were included in addition to the 15 studies identified from previous searches. A total of 19 studies (*n* = 19) were included in the review.

### Characteristics of included studies

Nineteen studies published between 2009 and June 2023 were included in the review. A combined overall sample size of 2,032,200 patients were included across all included studies. Included studies varied in the numbers of women with SBC for whom data could be extracted. Eight (*n* = 8) studies included women with secondary breast cancer exclusively [8, 29–35]. The remaining eleven studies (*n* = 11) included subgroups and cohorts of women with a secondary diagnosis [36–42, 42, 44–46]. These ranged from 3 to 21% of the overall sample. In total, 276,311 women with secondary breast cancer were included in the analysis and narrative synthesis. Sixteen studies (*n* = 16) were retrospective cohort design, two cross-sectional study (*n* = 2) and one descriptive survey design (*n* = 1). Thirteen studies (*n* = 13) were conducted in the USA and the remainder across Argentina, Brazil and Turkey. Primary data source for the US studies was either population-based databases or cancer registries (Table 1).

**Table 1:** Characteristics of included studies (*n* = 19)

Author (year)	Sample size/no. of SBC patients included (%)	Study design	Country	Study population	SACT	Data source	Primary exposure	Exposures/covariates	Outcome of interest	Statistical analysis
Accordino et al. (2017) [26]	4251/4521 (100)	Retrospective cohort	USA	Women > 66, confirmed stage IV diagnosis between January 2002–2011, who died by 31 December 2012	Chemotherapy	SEER-Medicare	Time from diagnosis to death, costs of care, location of death	Age, year of diagnosis, marital status, race, hospital location (urban/rural), geographic region, comorbidities, HR status, No. of consultations	Receipt of end-of-life (EoL) care which included receipt of IV chemotherapy within 14 days of death	Logistic regression model to determine association between clinical, demographic and prognostic factors and receipt of EoL care
Alves et al. (2022) [33]	296/2525 (12)	Analytical cross-sectional study	Brazil	Patients with a confirmed BC diagnosis and without treatment from January to December 2019	Receipt of no SACT treatment	Cancer Hospital Registers (CHR)	-	Age group, race education, marital status, smoking status, alcohol status, region, family history, referral source, tumour, stage at diagnosis	Lack of access to BC treatment	Multivariate regression for factors associated with lack of access to SACT. odds ratio (OR) values with a 95% confidence interval (CI 95%), and estimated *p*-values
Cole et al. (2019) [34]	65,380/601,680 (11)	Retrospective cohort	USA	Patients > 40 years with metastatic prostate, lung, colon and breast cancer, diagnosed from January 1, 2004, to December 31, 2015	Chemotherapy	NCDB	Minority serving hospital (top decile) (MSH)	Age, race, year of diagnosis, insurance status, education income, comorbidities	Receipt of specialist palliative care (SPC) which included non-curative systemic chemotherapy	Multilevel logistic regression model which estimated odds of palliative care, adjusted for year of diagnosis, sex, race/ethnicity, insurance, income, educational level and cancer type
Falchook et al. (2017) [35]	5855/28,731 (20)	Retrospective cohort	USA	Patients > 18 < 64 years at time of death, who died between 1 Jan 2007 and 31 Dec 2014, with metastatic lung, colorectal, breast, pancreatic or prostate cancer with a diagnosis code reflecting metastatic disease during the 12 months preceding death	Chemotherapy	HIRD	Not specified	Age, year of death, population density, geographical region	Receipt of end-of-life (EoL) care which included chemotherapy in the last 14 days of life	Modified Poisson regression models to estimate risk for each outcome, adjusted for age, sex, geographic region, rural/urban location, year of death and regional education and income measures
Ferreira et al. (2020) [36]	10,816/151,931 (7)	Cross sectional	Brazil	Women > 18 > 70 diagnosed with breast cancer between 1998 and 2012	Unspecified SACT	NCI HRC	Not specified	Referral route, stage, insurance status, family history, time between diagnosis and treatment, geographic region, marital status, education, race, age	Time between diagnosis and commencement of treatment < 60 days or > 60 days	Logistic regression of time between diagnosis and treatment adjusted for clinical and epidemiologic characteristics. Addition and removal of variables in the model with significance levels for the removal and addition of variables in the models were *p* ≤ 0.20 and *p* > 0.05, respectively
Giap et al. (2023) [27]	60,685/60,685 (100%)	Retrospective cohort	USA	Patients aged > 18 years diagnosed with de novo stage IV breast cancer between 2010 and 2017	Chemotherapy (non-curative)	NCDB	Race and ethnicity	Facility type, income, insurance, education, residential setting, diagnosis age, comorbidities, diagnosis year, tumour grade, metastasis location, tumour receptor types, prior treatment(s)	Receipt of palliative care which included non-curative systemic therapy	Multivariable logistic regression analysis to identify variables associated with receipt of palliative care
Ozmen et al. (2015) [37]	29/1031 (3)	Questionnaire survey	Turkey	Women aged ≥ 18 years diagnosed with breast cancer within 6 months prior to questionnaire completion and undergoing/preparing for treatment	Unspecified SACT	Validated questionnaire	Not specified	Patient characteristics, symptom detection, first admission public/private hospital, surgical treatment, stage, lymph node involvement	Time to receipt of treatment defined as follows: (i) patient delay time (PDT), (ii) system delay time (SDT), and (iii) total delay time = sum of PDT and SDT	Chi-square tests and one-way analysis of variance (ANOVA) for group differences, principal components analysis for variable reduction and multiple regression analysis to build predictive models of PDT, SDT and TDT
Recondo et al. (2019) [38]	268/13 (5)	Retrospective cohort	Argentina	Patients ≥ 18 years old with diagnosis of non-small cell lung cancer (NSCLC) or breast cancer, stages I to IV treated with chemotherapy from January 1, 2016	Chemotherapy	Participant medical records	Not specified	Nationality, civil status, income, employment, transportation, travel time, stage, detection, performance status, treatment modality, chemotherapy provider	Time elapsed between diagnosis and receipt of treatment with chemotherapy	Kaplan–Meier curves were used to estimate patterns in time to diagnosis and to initial treatment and compared by the log rank test. Cox proportional hazard to explore association between health system and individual and sociodemographic and clinical variables
Sathe et al. (2023) [28]	6802/6802 (100%)	Retrospective cohort	USA	Patients ≥ 18 years old, diagnosed with HR-positive, HER2-negative MBC between February 3, 2015 (first FDA approval of a CDK4/6i) and July 31, 2021	Cyclin-dependent kinase 4 and 6 (CDK4 and CDK6) inhibitors	Flatiron health database (FHD)	Race	Age at MBC diagnosis, insurance coverage, site of treatment, performance status, year of metastatic diagnosis, the presence or absence of baseline leukopenia	Receipt of cyclin-dependent kinase 4 and 6 (CDK4 and CDK6) inhibitors palbociclib, ribociclib or abemaciclib documented in the FHD at any time after a MBC diagnosis	Multivariable logistic regression models were developed to analyse the association between CDK4/6i use and sociodemographic, clinical characteristics
Shih et al. (2009) [39]	42,804/207,581 (21)	Retrospective cohort	USA	Patients ≥ 18 years old newly diagnosed with metastatic breast cancer, colorectal cancer and NHL, who received immunotherapy treatment between 1998 and 2004	Immunotherapy	NCDB	Not specified	Age, race, ethnicity, year of diagnosis, insurance status, income, education, facility type	Receipt of immunotherapy following cancer diagnosis	Logistic regressions to examine factors associated with immunotherapy use in each of the three cancers. Separate analysis for the elderly and non-elderly samples to avoid the issue of near collinearity between age and insurance variables
Shiovitz et al. (2015) [40]	3583/76,259 (5)	Retrospective cohort	USA	Patients ≥ 18 years diagnosed with metastatic breast, colon, lung, prostate, ovarian and gastric cancers between 2001 and 2007	Chemotherapy	SEER-Medicare	Race (NHW/AI/Ans)	Age, cancer type, geographic region, year of diagnosis	Utilisation of cancer directed therapy which included chemotherapy	Logistic regression was used to estimate odds ratios (OR) and 95% confidence intervals (95% CI)
Statler et al. (2019) [30]	6234/6234 (100)	Retrospective cohort	USA	Patients 18 years or older diagnosed with stage IV (defined as metastatic to a distant site, M1 per American Joint Committee on Cancer TNM Staging Criteria), hormone receptor-positive (ER + and/or PR +) and HER2 + breast cancer who received treatment between 2010 and 2015	Not specified	NCDB	Not specified	Age, race/ethnicity, comorbidities, insurance status, income, facility type, distance to treatment, grade, sites of metastases and treatment	Treatment receipt for chemotherapy and hormone (endocrine) therapy	Multivariable logistic regression analyses were conducted to identify independent predictors of treatment receipt (chemotherapy vs. hormonal therapy)
Skinner et al. (2021) [32]	608/608 (100)	Retrospective cohort	USA	Women aged ≥ 18 years, diagnosed with metastatic triple negative breast cancer (mTNBC) between 1 January 2010 and 31 January 2016	Unspecified SACT	Vector Oncology Data Warehouse	Not specified	Age, race, BMI, Insurance, region, stage, performance, comorbidities, site of metastases	Treatment receipt (PFS, OS)	Logistic regression methods were used to evaluate predictors of receipt of systemic anticancer therapy
Small et al. (2012) [41]	57,148/773,233 (7)	Retrospective cohort	USA	Patients > 18 diagnosed with stage IV cancer of kidney, uterus, NSCLC, SCLC, rectum, colon, prostate, breast and cervix between 2000 and 2008 who did not receive any first‐course therapy	Unspecified SACT	NCDB	Not specified	Age, race, ethnicity, insurance status, education, income	Receipt of no first course anticancer therapy	Log‐binomial regression to estimate prevalence ratios (PRs) with 95% confidence limits (95% CL) relating the percentages of patients receiving no first‐course therapy versus any therapy for stage IV cancer among categories of various socioeconomic and demographic variables. Statistical significance was defined as *p* < .05
Vas Luiz et al. (2015) [31]	4364/4364 (100)	Retrospective cohort	USA	Women > 66 with a first invasive metastatic breast cancer diagnosed between October 1998 and December 2009 enrolled in Parts A/B Medicare	Immunotherapy	SEER-Medicare	Race (White/Black)	Age, marital status, income, education, year of diagnosis, location of residence (metro/nonmetro), geographical location (SEER region), comorbidity, grade, HR status	Time to trastuzumab initiation and utilisation of trastuzumab	Wilcoxon signed-rank tests and multivariable linear regression to examine utilisation adjusting for exposure variables stratified by HR status
Vyas et al. (2021) [5]	1089/1089 (100%)	Retrospective cohort	USA	Women aged > 65 years diagnosed with HER2-negative MBC during 2010–2013	Unspecified SACT	SEER-Medicare	Not specified	Predisposing characteristics comprised age at diagnosis, race/ethnicity, whilst enabling characteristics included marital status, household income and education Need-related factors included HR status, grade of tumour, comorbidity scores, performance status and number of sites with cancer metastasis External care environmental factors comprised the location of residence, SEER region and census level information on the number of hospitals offering oncology services	Receipt of guideline-concordant initial treatment within the first 6 months of a cancer diagnosis	Multivariable logistic regression to identify the significant predictors of guideline-concordant treatment and a non-linear decomposition method to examine disparities by HR status
Wan & Jubelirer (2015) [35]	4533/4533 (100)	Retrospective cohort	USA	Women > 66 diagnosed with stage IV metastatic breast cancer between 1992 and 2002	Chemotherapy	SEER-Medicare	Not specified	Age, race, ethnicity, year of diagnosis, income, marital status, HR status, comorbidities, rural/urban, geographical location (SEER region), area chemotherapy rate, travel time, oncologist provision, hospice provision	Receipt of chemotherapy within 6 months (183 days) of diagnosis	Multivariate logistic regression aggregate models with interaction terms and subgroup analyses
Wang & Du (2015) [42]	1100/25,128 (4)	Retrospective cohort	USA	Women > 65 diagnosed with hormone receptor-positive breast cancer aged between 2006 and 2009 with Part-D claims up to December 2010	Hormone therapy	SEER-Medicare	Not specified	Age, race, marital status, comorbidity, SES, geographical location (SEER region), location of residence (urban/rural), year of diagnosis, stage, grade, lymph node involvement	Receipt of hormone therapy and overall use of hormone therapy 1 year post initiation (stratified by chemotherapy use)	Multivariate logistic regression assessed variation associated with the use of hormone therapy, SERM and AIs, respectively, adjusted for age, gender, race, marriage status, SEER registry area, year of diagnosis, tumour stage, tumour size, radiation therapy status, surgery status and comorbidity
Wolfson et al. (2015) [43]	1441/75,987 (4)	Retrospective cohort	USA	Patients aged > 22 < 65 with newly diagnosed adult-onset breast, cervical colorectal, gastric, hepatobiliary, lung, oral, and pancreatic cancer between 1998 and 2008	Unspecified SACT	NCI LAC	Not specified	Age, race/ethnicity, SES, distance to nearest NCI CCC	Receipt of care which included including SACT at National Cancer Institute Comprehensive Cancer Centre (NCI CCC)	Logistic regression analysis for multivariate modelling of likelihood of receiving care at an NCICCC. Two-sided tests with *p* < 0.05 were considered statistically significant

**Fig. 1:**
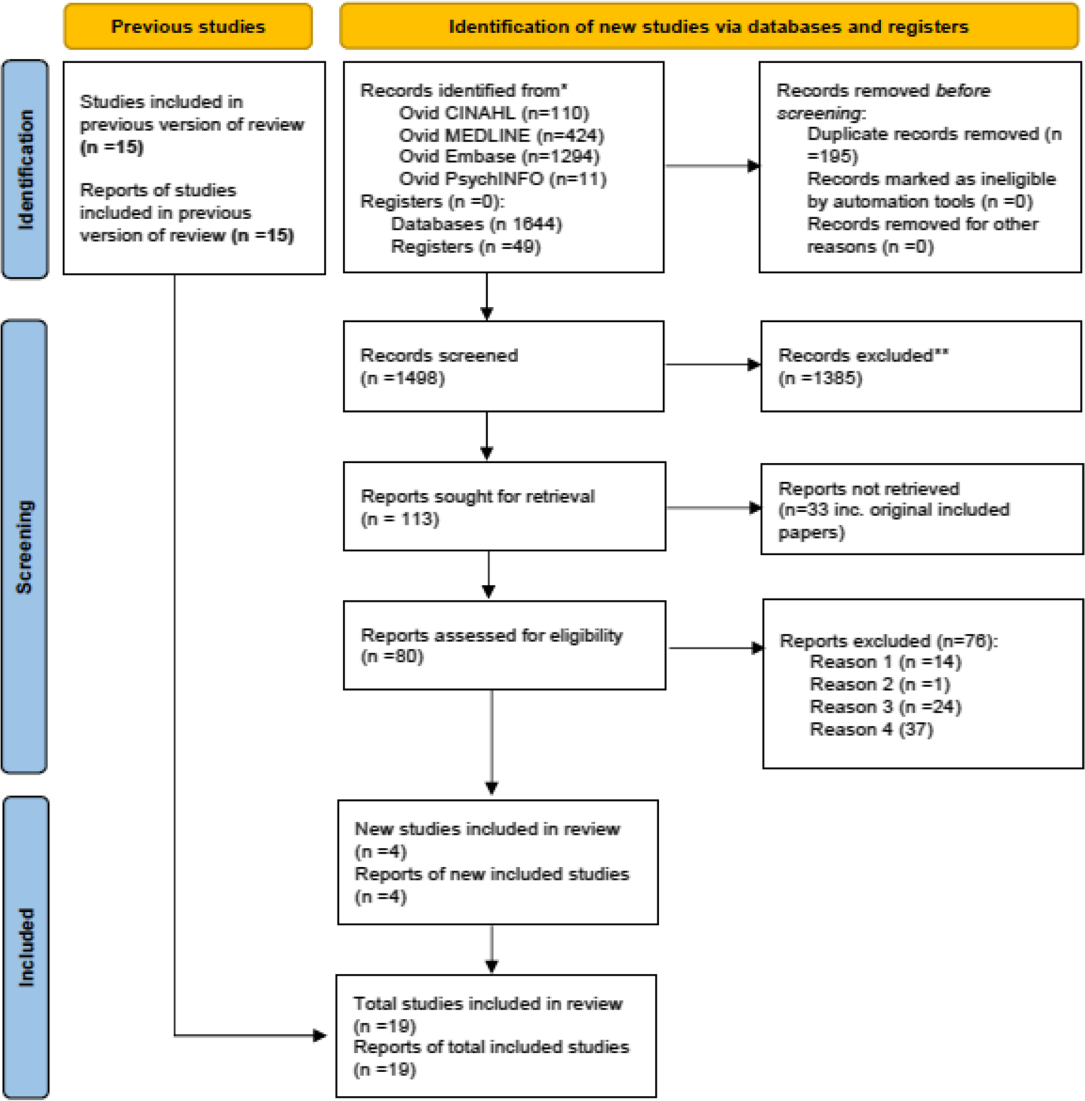
Completed PRISMA 2020 flow diagram for updated systematic reviews

All patients across all studies were aged > 18, and of those studies which included exclusively women with SBC, all were aged > 66. There was considerable variation across included studies in reported factors (exposures) associated with treatment receipt and time to treatment. These included demographic variables, i.e. age, race, socioeconomic status, income and education. Reported clinical characteristics included comorbidities, hormone receptor status and year of diagnosis. Contextual variables included geographical location, population density and place of care. Reported outcomes varied across studies, and whilst all studies reported treatment receipt or nonreceipt as either primary or secondary outcomes, measures of treatment receipt differed across studies. These included treatment receipt as a dichotomous outcome, i.e. received/not received, time to treatment as a continuous outcome and treatment received within the context of end-of-life (EoL) care and receipt of no first-line therapy. Characteristics of included studies are presented in Table 1 and Fig. 1.

### Assessment of methodological quality

Overall methodological quality was assessed as low to moderate. Levels of agreement between author and second reviewer were rated as moderate to high. Clear inclusion and exclusion criteria were specified (with the exception of two studies) which made it possible to determine study population and any differences within and across groups. There were considerable differences across studies in relation to assessment and measurement of exposures. Identification, assessment and management of confounding variables was assessed to determine how this was addressed in the statistical analysis. This was adequately addressed in the majority of studies; however, confounding variables included in analyses varied across studies which made for challenges in comparison across studies. Studies were assessed to determine validity and reliability of outcome measure, and all studies demonstrated this. Grading of Recommendations, Assessment, Development and Evaluations (GRADE) rating was assessed as low to moderate across studies [26]. Based on the assessment of methodological quality, GRADE rating and proportion of secondary breast cancer patients included in each study were attributed a weighting which was reflected in the analysis and synthesis (Table 2).

**Table 2:** Assessment of methodological quality including proportion of secondary breast cancer patients included, quality rating, GRADE rating and overall study weighting (*n* = 19)

No	Author (year)	Sample size (% of SBC patients) included	Quality rating^a^	GRADE rating^b^	Overall study weighting^c^
1	Accordino et al. (2017) [26]	4251/4521 (100)	Moderate	Moderate	1
2	Alves et al. (2022) [33]	296/2525 (12%)	Low	Low	3
3	Cole et al. (2019) [34]	65,380/601,680 (11)	Moderate	Moderate	2
4	Falchook et al. (2017) [35]	5855/28,731 (20)	Moderate	Moderate	2
5	Ferreira et al. (2020) [36]	10,816/151,931 (7)	Low	Low	3
6	Giap et al. (2023) [27]	60,685/60,685 (100)	Moderate	Moderate	2
7	Ozmen et al. (2015) [37]	29/1031 (3)	Low	Low	3
8	Recondo et al. (2019) [38]	268/13 (5)	Low–moderate	Low–moderate	3
9	Sathe et al. (2023) [28]	6082/6082 (100)	Moderate	Moderate	1
10	Shih et al. (2009) [39]	42,804/207,581 (21)	Moderate	Moderate	2
11	Shiovitz et al. (2015) [40]	3583/76,259 (5)	Moderate	Moderate	2
12	Skinner et al. (2021) [32]	608/608 (100)	Moderate	Moderate	2
13	Small et al. (2012) [41]	57,148/773,233 (7)	Moderate	Moderate	1
14	Statler et al. (2019) [30]	6234/6234 (100)	Moderate	Moderate	1
15	Vas Luiz et al. (2015) [31]	4364/4364 (100)	Moderate	Moderate	2
16	Vyas et al. (2021) [5]	1089/1089 (100)	Moderate	Moderate	1
17	Wan & Jubelirer (2015) [35]	4533/4533 (100)	Moderate	Moderate	1
18	Wang & Du (2015) [42]	1100/25,128 (4)	Low–moderate	Low–moderate	3
19	Wolfson et al. (2015) [43]	1441/75,987 (4)	Low–moderate	Low–moderate	3

^a^Joanna Briggs Institute assessment of methodological quality [24, 25]

^b^GRADE quality rating [26]. Low, true effect might be markedly different from the estimated effect. Moderate, true effect is probably close to the estimated effect

^c^Overall study weighting for contribution to narrative synthesis

1 = High

2 = Moderate

3 = Low

### Narrative synthesis

**Fig. 2:**
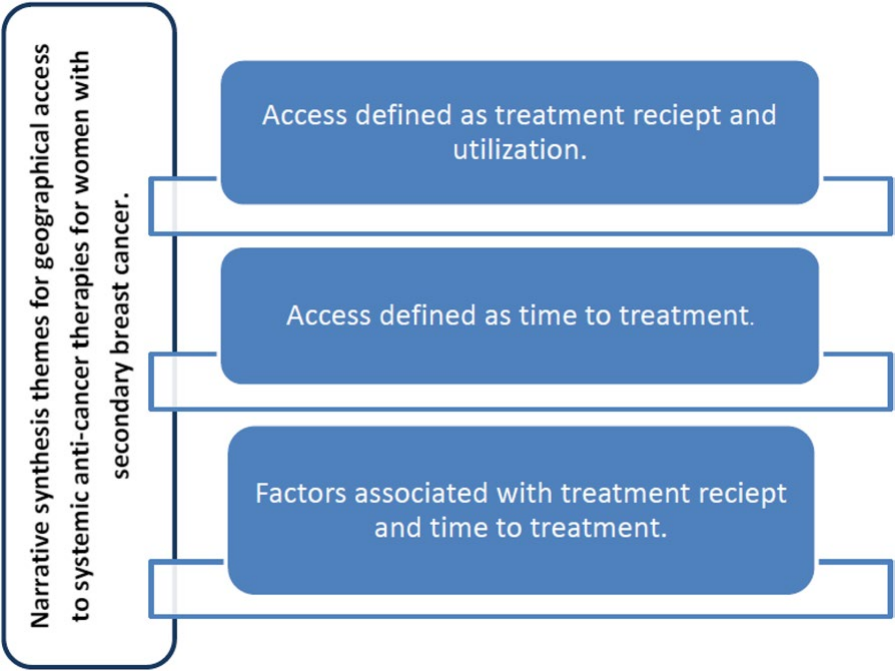
Narrative synthesis themes

The narrative synthesis reported key themes which were identified inductively from outcomes reported across included studies [47, 48]. Themes reflected review objectives to investigate factors associated with receipt of SACT for the treatment of SBC. The process used to conduct the thematic analysis reflected that proposed by Braun and Clarke [47] integrated with guidance on the conduct of narrative synthesis in systematic reviews presented by Popay et al. [28]. Included studies were reviewed, data extracted, and themes identified inductively based on reported outcomes across included studies. The narrative syntheses were structured around included studies which reported key themes for treatment receipt and utilisation, time to treatment and factors associated with time to treatment and treatment utilisation. This provided a framework for the overarching synthesis (Fig. 2).

Themes were identified for the following:

- Access to treatment as defined by receipt and/or treatment utilisation
- Access to treatment defined as time to receipt of treatment/treatment utilisation
- Factors associated with access to treatment (treatment receipt/utilisation and/or time to receipt of treatment/utilisation

### Access to treatment defined as treatment receipt and utilisation

Fifteen studies reported treatment receipt. Treatment receipt was reported for different types of SACT which included chemotherapy, immunotherapy, targeted therapy which included cyclin-dependent kinase 4 and 6 inhibitors (CDK4/6i), unspecified SACT and endocrine therapy. Seven studies reported chemotherapy where overall receipt ranged from 22 to 60% [29, 30, 35, 37, 38, 41, 43]. Receipt of endocrine therapy was reported in one study which evaluated factors associated with treatment receipt among women with hormone receptor-positive disease where overall treatment receipt was reported as 70.8% [45]. Receipt of immunotherapy reported in two studies was 4% [34, 42]. Six studies reported treatment receipt with SACT which was unspecified. Two studies reported receipt of no-treatment women who did not receive a first course of treatment; this ranged from 11.1 to 25.5% with 12.8% of women with a breast cancer diagnosis who did not receive a first course of treatment [36, 44] (Table 3).

**Table 3:** Included studies which reported access to treatment defined as treatment receipt and utilisation (*n* = 15)

**Author (year)**	**Outcome of interest**	**Type of SACT treatment**	**Main finding(s)**
Accordino et al. (2017) [26]	Receipt of EOL care which included receipt of IV chemotherapy within the last 14 days of life	IV chemotherapy as treatment within the last 14 days of life	60% receipt of IV chemotherapy as treatment within the last 14 days of life
Alves et al. (2022) [33]	Receipt of no SACT treatment	Unspecified SACT	60% of women received no first-course treatment
Cole et al. (2019) [34]	Receipt of specialist palliative care (SPC) which included non-curative systemic chemotherapy	Non-curative systemic chemotherapy	18.5% received specialist palliative care (SPC) which included non-curative systemic chemotherapy
Falchook et al. (2017) [35]	Receipt of chemotherapy within the last 14 days of life	Chemotherapy within the last 14 days of life	14.1% received chemotherapy within the last 14 days of life
Giap et al. (2023) [27]	Receipt of palliative care which included non-curative systemic therapy	Non-curative systemic chemotherapy	21.4% received non-curative systemic chemotherapy
Sathe et al. (2023) [28]	Receipt of cyclin-dependent kinase 4 and 6 (CDK4 and CDK6) inhibitors following secondary (metastatic) breast cancer diagnosis	Cyclin-dependent kinase 4 and 6 (CDK4 and CDK6) inhibitors	76.3% received CDK4/6i
Shih et al. (2009) [39]	Receipt of immunotherapy post diagnosis	Immunotherapy	4% received immunotherapy post diagnosis
Shiovitz et al. (2015) [40]	Utilisation of cancer-directed therapy which included chemotherapy	Chemotherapy	61% received SACT
Skinner et al. (2021) [32]	Receipt of systemic anticancer therapy	Unspecified SACT	83% received SACT
Small et al. (2012) [41]	Receipt of no first course anticancer therapy	Unspecified SACT—no first-course therapy	12.8% received no first-course SACT therapy
Statler et al. (2019) [30]	First-line treatment with chemotherapy and/or hormone therapy	First-line treatment with chemotherapy and/or hormone therapy	60% received first-line treatment with hormone therapy, 39% received chemotherapy, and 42% of patients received anti-HER2 therapy
Vyas et al. (2021) [5]	Receipt of initial systemic therapy within 6 months of secondary (metastatic) breast cancer diagnosis	Unspecified systemic anticancer therapy	72.3% received initial (guideline concordant) systemic therapy within 6 months of secondary (metastatic) breast cancer diagnosis
Wan & Jubelirer (2015) [35]	Receipt of chemotherapy within 6 months of diagnosis	Chemotherapy	30.2% received chemotherapy
Wang & Du (2015) [42]	Receipt of hormone therapy 1 year post initiation (stratified by chemotherapy use)	Hormone (endocrine) therapy	70.8% received hormone therapy
Wolfson et al. (2015) [43]	Receipt of care which included un specified SACT SACT at a National Cancer Institute Comprehensive Cancer Centre (NCI CCC)	Nonspecified SACT	**22–39 years 9.7% 40–65 years 90.3%

### Time to treatment

Four studies reported time to treatment [34, 39–41]. These included two studies which reported time to treatment with non-specified SACT chemotherapy and immunotherapy [39, 40]. Overall, time to treatment ranged from 54 days (median) *IQR* 32–124 (unadjusted) for White patients compared with 95 days *IQR* 39–167 for non-White (unadjusted) < 0.05 [34]. Patient delay time was reported as 4.8 weeks with a total delay time of 13.8 weeks [40]. Eighty-one percent of patients received treatment within 60 days [39], and treatment delay differences between public and private hospitals were reported as 76 days (64–86) < 0.0001 and 60 days (52–65) < 0.0001 respectively [41] (Table 4).

**Table 4:** Table of studies which reported time to treatment as a narrative theme

Author (year)	Outcome	Narrative theme	Type of SACT	Time to treatment
Ferreira et al. (2020) [36]	Time between diagnosis and commencement of treatment > 60 days	Time to treatment	SACT (treatment)	< 60 days 81.3%
Ozmen et al. (2015) [37]	Time to receipt of treatment defined as (i) patient delay time (PDT), (ii) system delay time (SDT) and (iii) total delay time = sum of PDT and SDT	Time to treatment	SACT (treatment)	Patient delay time (PDT) 4.8 weeks System delay time (SDT) 10.5 weeks Total delay time (TDT) 13.8 weeks
Recondo et al. (2019) [38]	Time elapsed between diagnosis and receipt of treatment with chemotherapy	Time to treatment	Chemotherapy	Public hospitals 76 days (64–86) < 0.0001 Private hospitals 60 days (52–65) < 0.0001
Vas Luiz et al. (2015) [31]	Time to trastuzumab initiation and utilisation of trastuzumab	Time to treatment	Immunotherapy	White patients 54 days (median), *IQR* 32–124 (unadjusted) Non-White patients 95 days, IQR 39–167 (unadjusted) < 0.05

### Factors associated with treatment receipt and time to treatment

#### Sociodemographic (individual) factors

Age was the most widely reported demographic across studies. For studies which reported adjusted odds ratio (OR), older age was associated with a reduced likelihood of treatment receipt and an increase in delay to treatment for chemotherapy, immunotherapy, unspecified SACT and hormone therapy [34, 39–41]. For palliative chemotherapy, older women (> 81 years) had a lower likelihood of receipt than those aged < 50 years (reference group) (*OR* 0.79 0.77–0.82) [37]. Receipt of treatment with immunotherapy was less likely for those in the oldest age quartile compared with those in the youngest (reference group) (Q1 *OR* 0.28 0.19–0.42) [42]. Older women had a lower likelihood of receipt of CDK4/6i; in particular, women aged > 75 were almost half as likely as younger women for overall use and 20% lower likelihood of first-line use (*OR* 0.54 0.45–0.64 and *OR* 0.84 0.72–0.98) respectively [31].

For hormone therapy, likelihood of receipt reduced with increasing age (*OR* 0.49 0.33–0.71 for those aged 85–89 compared with *OR* 0.78 0.63–0.97 for those aged 75–79) [45]. Older age was associated with reduced likelihood of treatment receipt at an NCI CCC with older women less likely than younger women to receive treatment (*OR* 0.7 (0.6–0.8), *P* < 0.001) [46]. These findings suggested that older women were less likely to receive treatment than younger women. Older age was associated with increased likelihood of receipt of no first-course treatment (36.44), and older women were less likely than younger women to receive SACT as part of end-of-life (EoL) care [29].

Lower likelihood of treatment receipt associated with race was reported with non-White patients less likely to receive treatment and in a less timely manner compared to White counterparts [37, 42, 45, 46]. Asian patients were least likely to receive treatment with palliative chemotherapy compared with White, Black and Hispanic women (*OR* 0.93 (0.88–0.98)) [37]. These findings were consistent for receipt of hormone therapy, specifically for receipt of SERMs where non-White patients had a lower likelihood of receipt (*OR* 0.62 (0.43–0.89)) and Black and Hispanic women less likely than White women to receive treatment at NCI CCC (*OR* 0.5 and 0.7) respectively [45, 46]. Conversely, non-White women were more likely to receive treatment as part of EoL care than white women and were more likely to receive no first-course therapy [29, 37].

For time to treatment and duration of treatment race and ethnicity demonstrated statistically significant difference between 344 days with 61% completion of treatment course for White women compared with 191 days and 44% completion for non-White women, *P* < 0.005 [34]. Socioeconomic status (SES) was reported across four studies where lower SES was associated with reduced likelihood of treatment receipt [42, 45, 46]. For hormone therapy, those with lower SES were less likely to receive treatment (*OR* 0.80 0.64–0.98). However, this was for receipt of AIs only [45]. Women with a lower SES were less likely to receive treatment at NCI CCC than women with higher SES (*OR* 0.3 (0.3–0.4), *P* < 0.001) [46].

#### Clinical characteristics

Eighteen studies reported clinical characteristics. These included year of diagnosis, comorbidities, hormone receptor status, stage and grade. Year of diagnosis ranged from 1992 to 2015 and was reported across seven studies. In two studies, those diagnosed in later years were more likely to receive palliative chemotherapy and immunotherapies respectively [37, 42]. Twelve studies reported comorbidities. Two studies reported a statistically significant association between fewer comorbidities and increased likelihood of treatment receipt for chemotherapy and hormone therapy respectively [23, 32]. For chemotherapy, this was reported as a statistically significant difference in proportion of chemotherapy use between 32% for those with no comorbidities to 8% > 2 *P* < 0.0001. For hormone therapy (without prior chemotherapy), likelihood of treatment receipt decreased with increased comorbidities with *OR* 0.83 (0.75–0.92) and 0.74 (0.66–0.83) respectively where 0 was the reference category. The inverse was reported for aggressive EOL care with increased likelihood of treatment receipt for those with greater comorbidities (*OR* 1.20 (1.03–1.39) > 2, *OR* 2.00 (1.70–2.35)) where those with no comorbidities were the reference group [29]. Similarly, for receipt of palliative care which included non-curative systemic therapy for women with de novo metastatic disease, those with greater comorbidities had an increased likelihood of receipt of palliative care than women with no comorbidities (*OR* 1.27, 1.17–1.39) [30].

Hormone receptor status was reported across five moderate quality studies which reported data exclusively for patients with a secondary breast cancer diagnosis [8, 29, 30, 34, 35]. Two studies reported chemotherapy receipt and receipt of SACT respectively. Higher proportion of chemotherapy use was found in women with hormone receptor-negative disease, *P* < 0.001 [35]. The second reported a decreased likelihood of receipt of SACT for women with hormone receptor-negative disease (*OR* 0.25 (0.17–0.36)) [8].

Five studies reported tumour stage using the American Joint Committee on Cancer (AJCC) classification stages I–IV [35], three of which reported descriptive statistics only. One study reported almost 30% lower likelihood of treatment delay for stage IV diagnosis compared with stage I (*OR* 0.70 (0.65–0.75) < 0.001) however only included 7% SBC patients as a proportion of the overall sample [26]. The remaining study reported an almost threefold increase (*OR* 2.67 (1.52–4.67)) in treatment receipt with hormone therapy for those diagnosed with stage IV disease, however included only 4% SBC patients as part of the overall sample [45].

#### Contextual factors

Sixteen studies reported contextual factors which included geographical location of residence, population density and place of care. Health care system factors related to area chemotherapy rate, oncologist provision and hospitals offering oncology services were also reported. Geographical location was reported across nine studies. This was generally classified at US regional level based on Surveillance, Epidemiology and End Results (SEER) programme of cancer registries classification of geographical region. Statistically significant differences in treatment receipt were reported between different geographical regions both within and across studies. Studies reported geographical differences in treatment receipt, receipt of systemic and cancer therapies as part of EoL care and time to treatment. Statistically significant associations between geographical region and treatment receipt were reported for EOL care with lower likelihood for those residing in the West (*OR* 0.60 (0.50–0.71)) compared with the East as the reference category. Despite heterogeneity across studies, these findings may suggest regional variation in treatment receipt and time to treatment.

Population density was reported across seven studies. Classification was reported using urban/rural/metropolitan/nonmetropolitan classification. The greatest proportion of patients resided in urban areas > 80% with the only statistically significant finding reported for aggressive EOL care where rural hospital location was associated with a reduced likelihood of aggressive care (*OR* 0.75 (0.60–0.93)) [29]. Geographical location was associated with treatment receipt; however, this was not an explicit association with specific geographical regions, rather variation between regions, which suggested a degree of regional variation in treatment receipt and time to treatment, though lacked specificity.

Seven studies reported place of care [29, 31, 33, 37, 41, 42, 46]. Measures included rural/urban hospital location [29], minority-serving hospital/nonminority-serving hospital (MSH/non-MSH) defined as top decile of hospitals serving minority patients [24], public/private hospitals [41] teaching/research/community and cancer centre [42] and NCI/non-NCI designated centre [46]. Patients treated at MSH (where non-MSH was the reference group) were almost 40% less likely to receive palliative chemotherapy [37]. Similarly, patients treated at community hospitals and cancer centres had a lower likelihood of treatment receipt across all age groups compared with patients treated at teaching and research hospitals (*OR* 0.52 (0.38–0.72), 0.68 (0.54–0.86), 0.6 (0.50–0.73) and 0.76 (0.67–0.87)) respectively [42]. Women who received treatment at an academic centre had an increased likelihood of receipt of CDK4/6i compared with women who received treatment at a nonacademic centre (*OR* 2.18 1.32–3.59) [31].

For patients treated at an NCI versus non-NCI designated centre, there were statistically significant differences in baseline characteristics specifically for race where a greater proportion of White patients were treated at NCI centres 7% NCI compared with 14% non-NCI, *P* < 0.001 [46]. Patients treated at rural hospitals had a reduced likelihood of receipt of aggressive EOL care (OR 0.75 (0.60–0.93)) compared with urban locations [29], and for time to treatment, those treated at public hospitals had greater median time between diagnosis to treatment compared with those treated at private hospitals 76 days (64–86) compared with 60 days (52–65) [41]. Only the first three studies exclusively reported SBC patients; however, these consistent findings would suggest an association between place of care and treatment receipt [29, 41, 42].

## Discussion

To our knowledge, our review was the first of its kind to identify and investigate geographic and sociodemographic factors associated with timely receipt of SACT for women with SBC. Receipt of SACT was found to be variable and ranged from 4% for immunotherapy treatment to 76% for the newer CDK4/6i to 78% for endocrine therapy and 80% for unspecified SACT (Shi, Sathe, Wang, Skinner). Twelve percent of women received no first-course therapy (Alves, small). In terms of timeliness of treatment, overall median time to treatment ranged from 54 to 96 days with differences attributed to health care system delays, treatment receipt at public or private hospital and race and ethnicity.

We found geographical differences for treatment receipt. These were reported between geographical regions though this was not an explicit association with specific geographical regions rather variation between regions. This suggested a degree of regional variation though lacked specificity. Importantly, place of care was found to be associated with treatment receipt. Patients treated at minority serving hospitals, community hospitals and cancer centres had a lower likelihood of treatment receipt. Our findings suggested an association between place of care and treatment receipt.

Our findings for geographical variation reflected those in the health geography literature for the relationship between access and receipt of care and treatment and geographical location. It has long been recognised that important variations in access to health care and health outcomes have been associated with geography. In particular, distance to health care has been identified as one of the most important geographic features that has affected health outcomes and the geographical distribution of health care facilities which has affected utilisation through differential opportunities for access to services.

Evidence of disparity and variation in relation to geography has previously been reported for early breast cancer for radiotherapy, surgical treatment and late stage diagnosis [49–52]. A recent systematic review which examined differences in distance and travel time across multiple settings found evidence for an association between distance and poorer health outcomes which was preceded by a review of geographical variation in access to chemotherapy across multiple disease sites which found evidence for significant differences in chemotherapy utilisation related to cancer network geography with limited evidence for distance travelled [53, 54]. These reviews offered conflicting evidence for geographical disparity and neither explicitly reported on women with a secondary diagnosis. Further disparities within and between small area geographies have been reported though remained largely unexplained for women with a secondary diagnosis [10]. Our findings have provided new insights for women with a secondary diagnosis which require further exploration in terms of the complex interaction and potential effect modification between geographical location and health care systems factors.

Further factors associated with the timely receipt of SACT for women with SBC were found for sociodemographics for age, race, ethnicity and socioeconomic status. Older women, women of non-White race and women with a low SES were consistently reported to have a lower likelihood of timely treatment receipt. Clinical characteristics for comorbidities and clinical subtype of disease were associated with timely treatment receipt. Women with fewer comorbidities were more likely to receive treatment. A greater proportion of chemotherapy use was reported for women with hormone receptor-negative disease, and women with triple-negative disease were less likely to receive guideline concordant treatment.

Our findings were consistent with the wider breast cancer literature. Where it has been recognised, increasing age has been associated with a number of disparities. These have included lower screening rates, increased likelihood of advanced disease at diagnosis, reduced likelihood for treatment and opportunities for participation in clinical trials than younger women [55]. It has also been reported that older women are less likely to receive guideline concordant treatment, and the prevalence of ageist attitudes may impact the treatment that older women with breast cancer receive [56, 57]. Further systematic review evidence has suggested that older people are less likely to receive radical treatment disease [55].

Similarly for race and ethnicity, our review found that those of non-White origin were less likely to receive treatment in a less timely manner. This is consistent with the literature which has demonstrated previous disparities in relation to race and ethnicity [58]. This is both indirectly where race and ethnicity interact with other variables such as education, employment, housing and overall socioeconomic status and directly where these confounding variables have been subject to control and adjustment, through statistical analysis. This has been further reported in the literature for early breast cancer with the potentially confounding relationship between race and tumour and treatment heterogeneity [59].

Our findings provided new insights from the evidence for geographical disparities in the timely receipt of SACT for women with an SBC diagnosis which has previously remained unknown. We interpreted our findings within the context of a patient-centred framework for access [12]. This provided a useful interpretation of our review findings in terms of the impact of geographical location on dimensions of service accessibility related to availability and accommodation and the interface with women’s ability to seek and reach health care impacted by their sociodemographic and living environment. This offered a useful theoretical interpretation of our main findings.

### Limitations

A number of limitations were acknowledged primarily related to the evidence included in the review. High levels of heterogeneity existed within and between studies in terms of factors reported which were associated with treatment receipt and time to treatment. Outcomes for time to treatment were not defined and measured consistently, and there were differences in the definition and measurement of outcomes for treatment receipt. This presented a particular challenge in the synthesis of these studies. Differences in reported outcomes and how these were measured at an individual study level also presented challenges in synthesis and precluded statistical meta-analysis.

There was significant variation in individual studies in terms of the number and proportion of women included with an advanced secondary breast cancer diagnosis. This presented challenges in terms of consistent data extraction despite the use of a uniform approach as part of the review methodology. This also presented challenges for the synthesis of studies in particular the weighting applied to the narrative. Study quality was assessed as low to moderate which may have had an impact on the overall findings from a methodological perspective. Furthermore, included studies were drawn from international populations which may have affected generalisability given the context-specific nature of geographic access to health care.

The published review protocol was developed and reported to conduct a mixed-method review. However, no qualitative studies were identified for inclusion. On this basis, it was also not possible to address the objective to explore women and clinicians experience of access and treatment receipt for secondary breast cancer. This also necessitated amendment to the published protocol for assessment of methodological quality and risk of bias and data analysis and synthesis.

### Implications for practice, policy and future research

Our findings have a number of practical implications. Acknowledging the existence and understanding the complex nature of variation are fundamental to the development of appropriate strategies to address this in practice. Addressing unwarranted variation which has arisen from geographical disparity and place of care would require intervention at a strategic level from service commissioners and providers. This would need to consider the potential interaction and confounding relationship with wider social issues. A key consideration at all levels would be improvement in the availability and quality of routinely collected ‘real-world’ data to evidence the extent of variation. This could then be used in a more targeted way to support the development of initiatives aimed at reducing unwarranted variation. To address sociodemographic variation, a range of approaches may be required. These could include the development and piloting of targeted interventions to improve cultural competency for those groups most affected [60]. Clinical variation may require a range of strategies aimed at better engaging clinicians in the implementation of evidence-based treatment recommendations, guideline concordance and shared decision-making for preference sensitive care [61].

The review identified an overall lack of qualitative research into the experience of women and clinicians in terms of treatment access and receipt. This requires addressing with future research required in this area. Geographical variation requires further careful investigation which needs to address the interaction between geographical location and place of care. This is particularly important given recent policy initiatives for the greater centralisation of specialist cancer services and the recent parliamentary calls for a National Secondary Breast Cancer audit which will begin to identify and address some of the issues identified in the review [62].

The review also demonstrated a requirement for further epidemiological research using consistent, valid and reliable measures of exposure and outcome. This is required to provide a clear focus on the needs of women with a secondary diagnosis in relation to treatment receipt and will offer a more valid and reliable insight into specific issues for this group of women whose treatment needs have become increasingly complex. In particular, work to examine the role of clinical subtype of disease and treatment receipt as guideline concordance is guided by clinical subtype, and this is of particular importance in the era of personalised medicine.

## Conclusion

Our review was the first of its kind to identify and investigate the broad range of factors associated with timely receipt of SACT for women with SBC. We identified variation associated with geographical location and place of care. This was identified as a potential interaction effect which adds to the existing literature in this area and requires further exploration in future research and at a strategic and policy level. Findings should however be interpreted with a degree of caution due to the limitations identified. Further research is required to address these limitations.

### Supplementary Information

**Additional file 1.** Inclusion and exclusion criteria for studies included in the review.

**Additional file 2.** Updated search strategies for Ovid CINAHL, Ovid MEDLINE, Ovid Embase and Ovid PsycINFO (August 2023).

**Additional file 3.** Preferred Reporting Items for Systematic Reviews and Meta-Analyses for searching (PRISMA-S) checklist [23].

**Additional file 4.** Preferred Reporting Items for Systematic Reviews and Meta-Analyses for searching (PRISMA 2020) checklist [19].

## Data Availability

Not applicable.

## Abbreviations

AJCC: American Joint Committee on Cancer
EoL: End of life
GRADE: Grading of Recommendations, Assessment, Development and Evaluations
HER2: Human epidermal growth factor receptor 2
HR: Hormone receptor status
JBI: Joanna Briggs Institute
MSH: Minority-serving hospital
NCI: National Cancer Institute
NICE: National Institute for Health and Care Excellence
OR: Odds ratio
PRISMA: Preferred Reporting Items for Systematic Reviews and Meta-Analyses
PICO: Population, intervention, comparison and outcomes
RUCA: Rural-urban commuting area
SBC: Secondary (metastatic or advanced) breast cancer
SACT: Systemic anticancer therapies
SERMs: Selective estrogen receptor modulators
SES: Socioeconomic status

## References

[1] Cardoso F, Spence D, Mertz S, Corneliussen-James D, Sabelko K, Gralow J, Cardoso MJ, Peccatori F, Paonessa D, Benares A, Sakurai N, Beishon M, Barker SJ, Mayer M (2018). Global analysis of advanced/metastatic breast cancer: decade report (2005–2015). Breast (Edinburgh, Scotland).

[2] Palmieri C, Owide J, Fryer K (2022). Estimated prevalence of metastatic breast cancer in England, 2016–2021. JAMA Netw Open.

[3] Cardoso F, Paluch-Shimon S, Senkus E, et al. 5th ESO-ESMO international consensus guidelines for advanced breast cancer (ABC 5). Ann Oncol. 2020. 10.1016/j.annonc.2020.09.010. Accessed 17 July 2022. The Breast. 10.1016/j.annonc.2020.09.010 PMC7510449 32979513

[4] National Cancer Institute. Metastatic Cancer: When Cancer Spreads. 2020. https://www.cancer.gov/types/metastatic-cancer. Accessed 21 May 2022.

[5] National Institute for Health and Care Excellence: Managing Advanced Breast Cancer. 2017. https://www.nice.org.uk/guidance/cg81. Accessed 10 Oct 2020.

[6] Twelves C, Cheeseman S, Sopwith W (2020). Systemic treatment of hormone receptor positive, human epidermal growth factor 2 negative metastatic breast cancer: retrospective analysis from Leeds Cancer Centre. BMC Cancer.

[7] Rocque GB, Williams CP, Kenzik KM, Jackson BE, Azuero A, Halilova KI, Ingram SA, Pisu M, Forero A, Bhatia S (2018). Concordance with NCCN treatment guidelines: relations with health care utilization, cost, and mortality in breast cancer patients with secondary metastasis. Cancer.

[8] Vyas A, Mantaian T, Kamat S, Kurian S, Kogut S (2021). Association of guideline-concordant initial systemic treatment with clinical and economic outcomes among older women with metastatic breast cancer in the United States. J Geriatr Oncol.

[9] NHS England: Achieving World-Class Cancer Outcomes: A Strategy for England 2015–2020 Progress Report 2016–17*.* 2017. https://www.england.nhs.uk/wp-content/uploads/2017/10/national-cancer-transformation-programme-2016-17-progress.pdf. Accessed 3 June 2022.

[10] All Party Parliamentary Group on Breast Cancer: A Mixed Picture: An Inquiry into Geographical Inequalities and Breast Cancer. 2018. https://breastcancernow.org/get-involved/campaign-us/all-party-parliamentary-group-breast-cancer/report-geographical-inequalities-in-breast-cancer. Accessed 28 August 2021.

[11] Penchansky R, Thomas J. The Concept of Access: Definition and Relationship to Consumer Satisfaction. Medical Care. 1981. https://www.jstor.org/stable/3764310. 10.1097/00005650-198102000-00001 7206846

[12] Levesque J, Harris MF, Russell G (2013). Patient-centred access to health care: conceptualising access at the interface of health systems and populations. Int J Equity Health.

[13] Kitson, A., Marshall, A., Bassett, K. and Zeitz, K. What are the core elements of patient-centred care? A narrative review and synthesis of the literature from health policy, medicine and nursing. J Adv Nurs. 69: 4-15. 10.1111/j.1365-2648.2012.06064. 10.1111/j.1365-2648.2012.06064.x 22709336

[14] Institute of Medicine (US) Committee on Quality of Health Care in Americ (2001). Crossing the quality chasm: a new health system for the 21st century.

[15] Cu A, Meister S, Lefebvre B (2021). Assessing healthcare access using the Levesque’s conceptual framework– a scoping review. Int J Equity Health.

[16] Pearson SA, Taylor S, Marsden A (2021). Access to systemic anti-cancer therapies for women with secondary breast cancer—protocol for a mixed methods systematic review. Syst Rev.

[17] Lizarondo L, Stern C, Carrier J, Godfrey C, Rieger K, Salmond S, Apostolo J, Kirkpatrick P, Loveday H. Chapter 8: Mixed methods systematic reviews. In: Aromataris E, Munn Z (Editors). JBI Manual for Evidence Synthesis. 2020. Available from: https://synthesismanual.jbi.global. 10.46658/JBIMES-20-09. 10.11124/JBISRIR-D-19-00169 32813460

[18] Moher D, Shamseer L, Clarke M, Ghersi D, Liberati A, Petticrew M (2015). Preferred reporting items for systematic review and meta-analysis protocols (PRISMA-P) 2015: elaboration and explanation. BMJ.

[19] Page M J, McKenzie J E, Bossuyt P M, Boutron I, Hoffmann T C, Mulrow C D et al. The PRISMA 2020 statement: an updated guideline for reporting systematic reviews BMJ. 2021. 10.1136/bmj.n71. 10.1136/bmj.n71 PMC8005924 33782057

[20] Richardson WS, Wilson MC, Nishikawa J, Hayward RS. The well-built clinical question: a key to evidencebased decisions https://www.acpjournals.org/doi/10.7326/ACPJC-1995-123-3-A12. 10.7326/ACPJC-1995-123-3-A12 7582737

[21] Aromataris E, Munn Z (Editors). JBI Manual for Evidence Synthesis. JBI, 2020. Available from https://synthesismanual.jbi.global. 10.46658/JBIMES-20-01.

[22] Prady SL, Uphoff EP, Power M, Golder S. Development and validation of a search filter to identify equity-focused studies: reducing the number needed to screen. BMC Med Res Methodol. 18(1):106. 10.1186/s12874-018-0567-x. 10.1186/s12874-018-0567-x PMC6186133 30314471

[23] Rethlefsen ML, Kirtley S, Waffenschmidt S (2021). PRISMA-S: an extension to the PRISMA Statement for Reporting Literature Searches in Systematic Reviews. Syst Rev.

[24] Joanna Briggs Institute: Checklist for cohort studies. 2020. https://jbi.global/critical-appraisal-tools. Accessed 4 Aug 2020.

[25] Joanna Briggs Institute: Checklist for analytical cross sectional studies. 2020. https://jbi.global/critical-appraisal-tools. Accessed 4 Aug 2020.

[26] British Medical Journal: What is GRADE. 2023. https://bestpractice.bmj.com/info/toolkit/learn-ebm/what-is-grade/. Accessed 28 Aug 2023.

[27] Ryan R; Cochrane Consumers and Communication Review Group. Cochrane Consumers and Communication Review Group: data synthesis and analysis. http://cccrg.cochrane.org.

[28] Popay J, Roberts H, Sowden A, et al. Guidance on the Conduct of Narrative Synthesis in Systematic Reviews. https://www.lancaster.ac.uk/media/lancaster-university/content-assets/documents/fhm/dhr/chir/NSsynthesisguidanceVersion1-April2006.pdf.

[29] Accordino MK, Wright JD, Vasan S (2017). Association between survival time with metastatic breast cancer and aggressive end-of-life care. Breast Cancer Res Treat.

[30] Giap F, Ma SJ, Oladeru OT (2023). Palliative care utilization and racial and ethnic disparities among women with de novo metastatic breast cancer in the United States. Breast Cancer Res Treat.

[31] Sathe C, Accordino MK, DeStephano D, Shah M, Wright JD, Hershman DL (2023). Social determinants of health and CDK4/6 inhibitor use and outcomes among patients with metastatic breast cancer. Breast Cancer Res Treat.

[32] Skinner KE, Haiderali A, Huang M, Schwartzberg LS (2021). Real-world effectiveness outcomes in patients diagnosed with metastatic triple-negative breast cancer. Future Oncol.

[33] Statler AB, Hobbs BP, Wei W, Gupta A, Blake CN, Nahleh ZA (2019). Real-world treatment patterns and outcomes in HR+/HER2+ metastatic breast cancer patients: a national cancer database analysis. Sci Rep..

[34] Vaz-Luis I, Lin NU, Keating NL, Barry WT, Lii H, Winer EP, Freedman RA (2015). Racial differences in outcomes for patients with metastatic breast cancer by disease subtype. Breast Cancer Res Treat.

[35] Wan S, Jubelirer S (2015). Geographic access and age-related variation in chemotherapy use in elderly with metastatic breast cancer. Breast Cancer Res Treat.

[36] Alves MNT, Monteiro MFV, Alves FT, Dos Santos Figueiredo FW (2022). Determinants of lack of access to treatment for women diagnosed with breast cancer in Brazil. Int J Environ Res Public Health.

[37] Cole AP, Nguyen DD, Meirkhanov A, Golshan M, Melnitchouk N, Lipsitz SR, Kilbridge KL, Kibel AS, Cooper Z, Weissman J, Trinh QD (2019). Association of care at minority-serving vs non-minority-serving hospitals with use of palliative care among racial/ethnic minorities with metastatic cancer in the United States. JAMA Netw Open.

[38] Falchook AD, Dusetzina SB, Tian F, Basak R, Selvam N, Chen RC (2017). Aggressive end-of-life care for metastatic cancer patients younger than age 65 years. J Natl Cancer Inst.

[39] Ferreira N, Schoueri J, Sorpreso I, Adami F, Dos Santos Figueiredo FW. Waiting time between breast cancer diagnosis and treatment in Brazilian women: an analysis of cases from 1998 to 2012. Int J Environ Res Public Health. 2020. 10.3390/ijerph17114030. 10.3390/ijerph17114030 PMC7312631 32517042

[40] Ozmen V, Boylu S, Ok E, Canturk NZ, Celik V, Kapkac M, Girgin S, Tireli M, Ihtiyar E, Demircan O, Baskan MS, Koyuncu A, Tasdelen I, Dumanli E, Ozdener F, Zaborek P (2015). Factors affecting breast cancer treatment delay in Turkey: a study from Turkish Federation of Breast Diseases Societies. Eur J Pub Health.

[41] Recondo G, Cosacow C, Cutuli HJ, Cermignani L, Straminsky S, Naveira M, Pitzzu M, De Ronato G, Nacuzzi G, Taetti G, Corsico S, Berrueta M, Colucci G, Gibbons L, Gutierrez L, García-Elorrio E (2019). Access of patients with breast and lung cancer to chemotherapy treatment in public and private hospitals in the city of Buenos Aires. Int J Qual Health Care.

[42] Shih YC, Elting LS, Halpern MT (2009). Factors associated with immunotherapy use among newly diagnosed cancer patients. Med Care.

[43] Shiovitz S, Bansal A, Burnett-Hartman AN, Karnopp A, Adams SV, Warren-Mears V, Ramsey SD. Cancer-directed therapy and hospice care for metastatic cancer in american indians and alaska natives. Cancer Epidemiol Biomarkers. 2015. 10.1158/1055-9965.EPI-15-0251. 10.1158/1055-9965.EPI-15-0251 PMC4490969 25987547

[44] Small AC, Tsao CK, Moshier EL, Gartrell BA, Wisnivesky JP, Godbold JH, Smith CB, Sonpavde G, Oh WK, Galsky MD (2012). Prevalence and characteristics of patients with metastatic cancer who receive no anticancer therapy. Cancer.

[45] Wang X, Du XL. Socio-demographic and geographic variations in the utilization of hormone therapy in older women with breast cancer after Medicare Part-D coverage. Med Oncol. 201. 10.1007/s12032-015-0599-6. 10.1007/s12032-015-0599-6 25837434

[46] Wolfson JA, Sun CL, Wyatt LP, Hurria A, Bhatia S (2015). Impact of care at comprehensive cancer centers on outcome: Results from a population-based study. Cancer.

[47] Virginia Braun & Victoria Clarke (2006). Using thematic analysis in psychology. Qual Res Psychol.

[48] Thomas, J., Harden, A. Methods for the thematic synthesis of qualitative research in systematic reviews. BMC Med Res Methodology 8, 45. 10.1186/1471-2288-8-45. 10.1186/1471-2288-8-45 PMC2478656 18616818

[49] Bigby J, Holmes MD (2005). Disparities across the breast cancer continuum. Cancer Causes Control.

[50] Jacobs LK, Kelley KA, Rosson GD, et al. Disparities in Urban and Rural Mastectomy Populations. Ann Surg Oncol. 10.1245/s10434-008-0053-5. 10.1245/s10434-008-0053-5 18663535

[51] Tatalovich Z, Zhu L, Rolin A (2015). Geographic disparities in late stage breast cancer incidence: results from eight states in the United States. Int J Health Geogr.

[52] Mobley LR, Kuo TM, Watson L, Gordon BG (2012). Geographic disparities in late-stage cancer diagnosis: multilevel factors and spatial interactions. Health Place.

[53] Chamberlain C, Owen-Smith A, Donovan J (2016). A systematic review of geographical variation in access to chemotherapy. BMC Cancer.

[54] Kelly C, Hulme C, Farragher T, Clarke G (2016). Are differences in travel time or distance to healthcare for adults in global north countries associated with an impact on health outcomes? A systematic review. BMJ Open.

[55] Liverpool Reviews and Implementation Group (LRiG): Systematic review to examine the clinical effectiveness and tolerability of chemotherapy treatment for older people with colorectal cancer. 2015. https://www.liverpool.ac.uk/media/livacuk/iphs/systematic,review,chemotherapy,older,people,colorectal.pdf. Accessed 17 June 2021.

[56] Giordano SH, Hortobagyi GN, Kau SW, Theriault RL, Bondy ML (2005). Breast cancer treatment guidelines in older women. J Clin Oncol.

[57] The International Longevity Centre: Ageism in breast cancer. 2019. https://ilcuk.org.uk/ageism-in-breast-cancer. Accessed 20 June 2021.

[58] Reeder-Hayes KE, Troester MA, Wheeler SB. Adherence to Endocrine Therapy and Racial Outcome Disparities in Breast Cancer. The Oncologist. 10.1002/onco.13964. 10.1002/onco.13964 PMC8571754 34582070

[59] Tichy JR, Deal AM, Anders CK, Reeder-Hayes K, Carey LA (2015). Race, response to chemotherapy, and outcome within clinical breast cancer subtypes. Breast Cancer Res Treat.

[60] Truong M, Paradies Y, Priest N (2014). Interventions to improve cultural competency in healthcare: a systematic review of reviews. BMC Health Serv Res.

[61] The Kingsfund: Variations in health care: The good, the bad and the inexplicable. 2011. https://www.kingsfund.org.uk/publications/variations-health-care. Accessed 6 June 2021.

[62] Breast Cancer Now: Secondary counts – our secondary breast cancer campaign win. 2021. https://breastcancernow.org/about-us/news-personalstories/secondary-counts-%E2%80%93-our-secondary-breast-cancer-campaign-win. Accessed 28 May 2021.

