# Supplementary material for: Geographic and sociodemographic access to systemic anticancer therapies for secondary breast cancer: a systematic review
Source: Syst Rev. 2024 Jan 18;13:35. doi: 10.1186/s13643-023-02382-3 (PMC10795363; doi:10.1186/s13643-023-02382-3)
Supplement: Supplementary file 1 — Additional file 1. Inclusion and exclusion criteria for studies included in the review. [file 13643_2023_2382_MOESM1_ESM.docx]

**Additional file 1. Inclusion and exclusion criteria for studies included in the review:**

| **Study inclusion criteria:**  **Population:**  Studies reporting women >18 and with a confirmed SBC diagnosis who accessed/received treatment with SACT. This will include studies reporting all clinical sub types across all sites of metastases and will include de novo and recurrent presentations.  **Phenomena of interest/exposure(s):**  Studies which explore factors that are associated with access to SACT for SBC.  Studies reporting individual factors, which include, age, gender, sexual orientation, race/ethnicity, socioeconomic status, education, language and literacy, psychosocial characteristics. Studies reporting clinical characteristics which include, clinical sub type, HR status, HER2 status, previous treatment response and physician characteristics.  Contextual factors which include geography and geographical location, distance, travel time, health region, catchment/referral areas and organisational factors including, health care system factors, capacity, service availability.  **Comparator:**  Usual care  **Outcome:**  Studies which report access to, receipt and utilization of SACT.  **Type of study:**  Quantitative, qualitative and mixed methods studies. This will include observational, cross sectional, longitudinal and analytic studies, including, epidemiological studies, case control and cohort studies. Qualitative studies will include designs such as phenomenology, grounded theory, ethnography, action research and feminist research.  Mixed method studies will only be considered if data from the quantitative or qualitative components can be clearly extracted.  Studies published in English language, from January 2000 onwards, peer reviewed, original research, reporting quantitative, qualitative or mixed methods.  **Study exclusion criteria:**  **Population:**  Studies reporting primary and/or locally advanced (LABC) early stage (I-III) breast cancer only.  Studies reporting (comparative) treatment effect and efficacy.  Males with a secondary breast cancer diagnosis, as this is classified as a rare disease.  **Phenomena of interest/exposure(s):**  Studies which do not report individual, clinical and contextual factors associated with the primary outcome.  **Comparator:**  **Outcome:**  Studies which do not report the primary outcome measure of access to, and utilization of systemic anti-cancer therapies.  **Type(s) of study:**  Studies reporting (comparative) treatment effect and efficacy. Studies reporting clinical trials of systemic anti-cancer therapies, as whilst RCT is not a specific exclusion criterion it is likely that RCTs will measure treatment effect as opposed to access as an outcome and would therefore be excluded. |
| --- |
