# Supplementary material for: Geographic and sociodemographic access to systemic anticancer therapies for secondary breast cancer: a systematic review
Source: Syst Rev. 2024 Jan 18;13:35. doi: 10.1186/s13643-023-02382-3 (PMC10795363; doi:10.1186/s13643-023-02382-3)
Supplement: Supplementary file 2 — Additional file 2. Updated search strategies for Ovid CINAHL, Ovid MEDLINE, Ovid Embase and Ovid PsycINFO (August 2023). [file 13643_2023_2382_MOESM2_ESM.docx]

**Additional file 2. Updated search strategies for Ovid CINAHL, Ovid MEDLINE, Ovid Embase and Ovid PsycINFO (August 2023):**

**Search Strategy: Ovid CINAHL:**

| Search ID# | Search Terms | Results |
| --- | --- | --- |
| S1 | ( TI Secondary OR AB Secondary ) OR ( TI metastatic OR AB metastatic ) | View Results (238,419) |
| S2 | ( TI oncology or TI cancer or AB oncology or AB cancer) | View Results (521,869) |
| S3 | (MH "Neoplasms") | View Results (92,594) |
| S4 | TI breast* or TI mammary or AB breast* or AB mammary | View Results (149,464) |
| S5 | (MH "Breast Neoplasms") | View Results (95,384) |
| S6 | S2 OR S3 | View Results (547,217) |
| S7 | (S4 AND S6) OR S5 | View Results (123,935) |
| S8 | S1 AND S7 | View Results (12,980) |
| S9 | TI ( (sociodemographic or socioeconomic or ethnicity or race or psycho* or geograph* or location or distance or Neighbo?rhood* or rural* or inner?city or mortgage problem* or foreclosure or eviction or "housing loss" or overcrowding or Minorit* or Migration background or Racial or Racism or Ethnology or Race or Ethnic* or non?English or "Language other than" or Latino* or Latina* or Hispanic* or Whites or Caucasian* or non?white or Torres Strait Islander or Aboriginal or "Native American" or inuit or Eskimo or First Nation or Indigenous or "English as a second language" or "Foreign language" or occupations or unemployment or "Gender differences" or Gender identity or Sex Role or "Wom?n* Role?" or "M?n* Role? " or "Gender* Role?" or Servicewomen or Schooling or Educational status or Religi* or Disparit* or Inequalit* or inequit* or equity or Deprivation or Gini or Concentration index or SES or Disadvantaged or Impoverished or Poverty or Economic level or assets index or income* or Social exclusion or Social relationships or Social network* or Collective efficacy or Civil society or "Informal social control" or "Neighbo*rhood disorder" or Social Disorgani?ation or anomie or Trust or "Emotional support" or "Psychosocial support" or "Community Capital" or "Neighbo*rhood cohesion" or "Social Influence" or "Soci*context*" or "soci*-context*" or Health*care disparit* or "Health Care Disparit*" or "Health Status Disparit*" or "Health Disparit*" or "Health Inequalit*" or "Health Inequit*" or "Medically underserved") ) OR AB ( (sociodemographic or socioeconomic or ethnicity or race or psycho* or geograph* or location or distance or Neighbo?rhood* or rural* or inner?city or mortgage problem* or foreclosure or eviction or "housing loss" or overcrowding or Minorit* or Migration background or Racial or Racism or Ethnology or Race or Ethnic* or non?English or "Language other than" or Latino* or Latina* or Hispanic* or Whites or Caucasian* or non?white or Torres Strait Islander or Aboriginal or "Native American" or inuit or Eskimo or First Nation or Indigenous or "English as a second language" or "Foreign language" or occupations or unemployment or "Gender differences" or Gender identity or Sex Role or "Wom?n* Role?" or "M?n* Role? " or "Gender* Role?" or Servicewomen or Schooling or Educational status or Religi* or Disparit* or Inequalit* or inequit* or equity or Deprivation or Gini or Concentration index or SES or Disadvantaged or Impoverished or Poverty or Economic level or assets index or income* or Social exclusion or Social relationships or Social network* or Collective efficacy or Civil society or "Informal social control" or "Neighbo*rhood disorder" or Social Disorgani?ation or anomie or Trust or "Emotional support" or "Psychosocial support" or "Community Capital" or "Neighbo*rhood cohesion" or "Social Influence" or "Soci*context*" or "soci*-context*" or Health*care disparit* or "Health Care Disparit*" or "Health Status Disparit*" or "Health Disparit*" or "Health Inequalit*" or "Health Inequit*" or "Medically underserved") ) | View Results (984,137) |
| S10 | (MH "Ethnic Groups") | View Results (33,934) |
| S11 | (MH "Residence Characteristics") | View Results (15,082) |
| S12 | (MH "Marriage") | View Results (9,339) |
| S13 | (MH "Cultural Deprivation") | View Results (114) |
| S14 | (MH "Cultural Diversity") | View Results (16,164) |
| S15 | (MH "Language") | View Results (17,949) |
| S16 | (MH "Transients and Migrants") | View Results (5,869) |
| S17 | (MH "Emigration and Immigration") OR (MH "Immigrants") | View Results (23,296) |
| S18 | (MH "Minority Groups") | View Results (14,206) |
| S19 | (MH "Prejudice") | View Results (6,120) |
| S20 | (MH "Racism") OR (MH "Systemic Racism") | View Results (12,219) |
| S21 | (MH "Discrimination") | View Results (13,220) |
| S22 | (MH "Race Relations+") | View Results (12,955) |
| S23 | (MH "Refugees") | View Results (9,152) |
| S24 | (MH "Occupations and Professions") | View Results (5,195) |
| S25 | (MH "Unemployment") | View Results (5,293) |
| S26 | (MH "Gender Identity+") | View Results (9,649) |
| S27 | (MH "Women's Health") | View Results (46,925) |
| S28 | (MH "Sex Factors") | View Results (132,994) |
| S29 | (MH "Educational Status") | View Results (46,959) |
| S30 | (MH "Religion and Religions") | View Results (14,167) |
| S31 | (MH "Socioeconomic Factors") OR (MH "Low Socioeconomic Status") OR (MH "Socioeconomic Disparities in Health") | View Results (104,499) |
| S32 | (MH "Poverty") | View Results (27,290) |
| S33 | (MH "Stigma") | View Results (21,255) |
| S34 | (MH "Social Capital") | View Results (3,033) |
| S35 | (MH "Social Control") | View Results (2,761) |
| S36 | (MH "Support, Social") | View Results (3,719) |
| S37 | (MH "Social Environment") | View Results (13,021) |
| S38 | (MH "Trust") | View Results (14,223) |
| S39 | (MH "Social Status") | View Results (440) |
| S40 | (MH "Social Isolation") | View Results (11,359) |
| S41 | (MH "Social Participation") | View Results (6,771) |
| S42 | (MH "Health Status Disparities") | View Results (9,879) |
| S43 | (MH "Health Services Accessibility") | View Results (104,795) |
| S44 | TI ( (housing N2 (instability or insecurity or strain or security)) OR (home N2 (repossession* or ownership)) OR (repossess* N3 (hous* or propert*)) OR (Mortgage N2 (delinquency or arrears or debt*)) OR (living N1 (outside or inside or near* or Nacent)) OR (household N2 size) OR ((marital or marriage) N2 status) OR (widow* or cohabit* or divorce* or "single parent*" or "live* alone") OR ("sex disparit*" or "sex difference?") OR (Education* N2 level?) OR ((Higher or Better or Worse or Less) N2 educated) OR (Social N2 (determinants or status or position or background or circumstance*)) OR (socio-economic or socio-demographic) OR (Community N3 (cohes* or participa*)) OR ("social" N2 (support or participation)) OR ("Soci*context*" or "soci*-context*") ) OR AB ( (housing N2 (instability or insecurity or strain or security)) OR (home N2 (repossession* or ownership)) OR (repossess* N3 (hous* or propert*)) OR (Mortgage N2 (delinquency or arrears or debt*)) OR (living N1 (outside or inside or near* or Nacent)) OR (household N2 size) OR ((marital or marriage) N2 status) OR (widow* or cohabit* or divorce* or "single parent*" or "live* alone") OR ("sex disparit*" or "sex difference?") OR (Education* N2 level?) OR ((Higher or Better or Worse or Less) N2 educated) OR (Social N2 (determinants or status or position or background or circumstance*)) OR (socio-economic or socio-demographic) OR (Community N3 (cohes* or participa*)) OR ("social" N2 (support or participation)) OR ("Soci*context*" or "soci*-context*") ) | View Results (160,793) |
| S45 | S9 OR S10 OR S11 OR S12 OR S13 OR S14 OR S15 OR S16 OR S17 OR S18 OR S19 OR S20 OR S21 OR S22 OR S23 OR S24 OR S25 OR S26 OR S27 OR S28 OR S29 OR S30 OR S31 OR S32 OR S33 OR S34 OR S35 OR S36 OR S37 OR S38 OR S39 OR S40 OR S41 OR S42 OR S43 OR S44 | View Results (1,369,261) |
| S46 | TI ( Systemic OR (anti cancer or anticancer or sact) OR therap* OR “cancer drug” OR “hormone therapy” OR “endocrine therapy” OR “aromatase inhibitors” OR tamoxifen OR (oestrogen* or estrogen*) OR “epidermal growth factors” OR “biological therapy” OR “antibodies monoclonal” OR immunotherapy ) OR AB ( Systemic OR (anti cancer or anticancer or sact) OR therap* OR “cancer drug” OR “hormone therapy” OR “endocrine therapy” OR “aromatase inhibitors” OR tamoxifen OR (oestrogen* or estrogen*) OR “epidermal growth factors” OR “biological therapy” OR “antibodies monoclonal” OR immunotherapy ) | View Results (821,737) |
| S47 | (MH "Antineoplastic Agents") | View Results (54,015) |
| S48 | (MH "Drug Therapy") | View Results (15,931) |
| S49 | (MH "Aromatase Inhibitors") | View Results (2,123) |
| S50 | (MH "Tamoxifen") | View Results (4,116) |
| S51 | (MH "Epidermal Growth Factors") | View Results (5,968) |
| S52 | (MH "Biological Therapy") | View Results (2,353) |
| S53 | (MH "Antibodies, Monoclonal") | View Results (32,272) |
| S54 | (MH "Methotrexate") | View Results (6,841) |
| S55 | (MH "Immunotherapy") | View Results (14,319) |
| S56 | S46 OR S47 OR S48 OR S49 OR S50 OR S51 OR S52 OR S53 OR S54 OR S55 | View Results (887,384) |
| S57 | TI ( (Access or utili?ation or health care) ) OR AB ( (Access or utili?ation or health care) ) | View Results (602,366) |
| S58 | (MH "Health Care Delivery") | View Results (63,876) |
| S59 | S57 OR S58 | View Results (636,974) |
| S60 | S8 AND S45 AND S56 AND S59 | View Results (110) |
| S61 | S8 AND S45 AND S56 AND S59 Limiters - Published Date: 20000101-20231231 | View Results (108) |

**Search Strategy: OVID MEDLINE:**

| Ovid MEDLINE(R) ALL <1946 to August 23, 2023> | | |
| --- | --- | --- |
| # ▲ | Searches | Results |
| 1 | secondary.mp. | 1116761 |
| 2 | Neoplasm Metastasis/ or metastatic.mp. | 347868 |
| 3 | (oncology or cancer).mp. or Neoplasms/ | 2455972 |
| 4 | (breast* or mammary).mp. | 674915 |
| 5 | Breast Neoplasms/ | 331897 |
| 6 | 1 or 2 | 1381823 |
| 7 | (3 and 4) or 5 | 483288 |
| 8 | 6 and 7 | 80461 |
| 9 | sociodemographic.mp. | 73186 |
| 10 | socioeconomic.mp. | 259195 |
| 11 | ethnicity.mp. or Ethnic Groups/ | 148466 |
| 12 | race.mp. or Continental Population Groups/ | 160134 |
| 13 | psycho*.mp. | 2107609 |
| 14 | geograph*.mp. | 240629 |
| 15 | location.mp. | 361915 |
| 16 | distance.mp. | 300250 |
| 17 | Residence Characteristics/ | 38215 |
| 18 | Environment design/ | 7236 |
| 19 | exp Marital Status/ | 40211 |
| 20 | Neighbo?rhood*.mp. | 41749 |
| 21 | Residential Environment*.mp. | 968 |
| 22 | rural*.mp. | 205516 |
| 23 | inner?city.mp. | 56 |
| 24 | (housing adj2 (instability or insecurity or strain or security)).mp. | 1325 |
| 25 | mortgage problem*.mp. | 0 |
| 26 | foreclosure.mp. | 255 |
| 27 | eviction.mp. | 960 |
| 28 | housing loss.mp. | 26 |
| 29 | (home adj2 (repossession* or ownership)).mp. | 608 |
| 30 | (repossess* adj3 (hous* or propert*)).mp. | 8 |
| 31 | (Mortgage adj2 (delinquency or arrears or debt*)).mp. | 35 |
| 32 | overcrowding.mp. | 3862 |
| 33 | (living adj1 (outside or inside or near* or adjacent)).mp. | 4415 |
| 34 | (household adj2 size).mp. | 2040 |
| 35 | ((marital or marriage) adj2 status).mp. | 34901 |
| 36 | (widow* or cohabit* or divorce* or "single parent*" or "live* alone").ab,ti. | 26654 |
| 37 | Cultural deprivation/ or Acculturation/ or culture/ or cross-cultural comparison/ or cultural characteristics/ or cultural diversity/ or Language/ or "transients and migrants"/ or exp "emigrants and immigrants"/ or Minority groups/ or Minority health/ or Prejudice/ or Racism/ or Xenophobia/ or Social Discrimination/ or exp Race Relations/ or exp Ethnic Groups/ or exp Continental Population Groups/ or Refugees/ | 374043 |
| 38 | Minorit*.mp. | 106133 |
| 39 | Migration background.mp. | 890 |
| 40 | Racial.mp. | 77815 |
| 41 | Racism.mp. | 12079 |
| 42 | Ethnology.mp. | 174884 |
| 43 | Race.mp. | 144377 |
| 44 | Ethnic*.mp. | 223701 |
| 45 | non?English.mp. | 26 |
| 46 | Language other than.mp. | 682 |
| 47 | Latino*.mp. | 42938 |
| 48 | Latina*.mp. | 5571 |
| 49 | Hispanic*.mp. | 79344 |
| 50 | Whites.mp. | 30038 |
| 51 | Caucasian*.mp. | 70011 |
| 52 | non?white.mp. | 3211 |
| 53 | Torres Strait Islander.mp. | 2458 |
| 54 | Aboriginal.mp. | 10359 |
| 55 | Native American.mp. | 4882 |
| 56 | inuit.mp. | 4918 |
| 57 | Eskimo.mp. | 731 |
| 58 | First Nation.mp. | 540 |
| 59 | Indigenous.mp. | 45292 |
| 60 | English as a second language.mp. | 527 |
| 61 | Foreign language.mp. | 1871 |
| 62 | Occupations/ or unemployment/ | 32295 |
| 63 | occupations.mp. | 49323 |
| 64 | unemployment.mp. | 18006 |
| 65 | exp Gender Identity/ or women's health/ | 52977 |
| 66 | Gender differences.mp. | 33861 |
| 67 | ("sex disparit*" or "sex difference?").mp. | 48071 |
| 68 | Gender identity.mp. | 24629 |
| 69 | Sex Role.mp. | 1718 |
| 70 | Wom?n* Role?.mp. | 700 |
| 71 | M?n* Role? .ab,ti. | 11788 |
| 72 | Gender* Role?.mp. | 4158 |
| 73 | Servicewomen.mp. | 146 |
| 74 | Sex Factors/ or exp Educational Status/ or Education/ | 353195 |
| 75 | Schooling.mp. | 10423 |
| 76 | Educational status.mp. | 64159 |
| 77 | (Education* adj2 level?).mp. | 67447 |
| 78 | ((Higher or Better or Worse or Less) adj2 educated).mp. | 7449 |
| 79 | ((Higher or Better or Worse or Less) adj2 "Level? of education").mp. | 3509 |
| 80 | Religion/ | 15896 |
| 81 | Religi*.mp. | 73002 |
| 82 | Social determinants of health/ or psychosocial factors/ or sociological factors/ or working poor/ or hierarchy, social/ | 33788 |
| 83 | Disparit*.mp. | 126604 |
| 84 | Inequalit*.mp. | 50815 |
| 85 | inequit*.mp. | 21058 |
| 86 | equity.mp. | 33235 |
| 87 | Deprivation.mp. | 102120 |
| 88 | Gini.mp. | 2170 |
| 89 | Concentration index.mp. | 2225 |
| 90 | Socioeconomic Factors/ or Social Welfare/ or exp Social Class/ or exp Poverty/ or income/ | 276042 |
| 91 | (Social adj2 (determinants or status or position or background or circumstance*)).mp. | 37267 |
| 92 | (socio-economic or socio-demographic).mp. | 72944 |
| 93 | SES.mp. | 26673 |
| 94 | Disadvantaged.mp. | 17963 |
| 95 | Impoverished.mp. | 4236 |
| 96 | Poverty.mp. | 72337 |
| 97 | Economic level.mp. | 1430 |
| 98 | assets index.mp. | 24 |
| 99 | income*.mp. | 199340 |
| 100 | Social Stigma/ or social capital/ or social control, informal/ or exp social support/ or exp social environment/ or trust/ or social conditions/ or social isolation/ or social marginalization/ or anomie/ or social participation/ | 181843 |
| 101 | Social exclusion.mp. | 2661 |
| 102 | (Social adj3 (capital or cohes* or organis* or organiz*)).mp. | 15159 |
| 103 | (Community adj3 (cohes* or participa*)).mp. | 41722 |
| 104 | Social relationships.mp. | 7936 |
| 105 | Social network*.mp. | 27978 |
| 106 | Collective efficacy.mp. | 655 |
| 107 | Civil society.mp. | 2667 |
| 108 | Informal social control.mp. | 144 |
| 109 | Neighbo*rhood disorder.mp. | 306 |
| 110 | Social Disorgani?ation.mp. | 264 |
| 111 | anomie.mp. | 621 |
| 112 | ("social" adj2 (support or participation)).mp. | 116389 |
| 113 | Trust.mp. | 54546 |
| 114 | Emotional support.mp. | 8440 |
| 115 | Psychosocial support.mp. | 5751 |
| 116 | Community Capital.mp. | 31 |
| 117 | Neighbo*rhood cohesion.mp. | 238 |
| 118 | Social Influence.mp. | 2863 |
| 119 | ("Soci*context*" or "soci*-context*").ab,ti. | 14456 |
| 120 | Health Status Disparities/ or Health Services Accessibility/ or Health Equity/ | 106665 |
| 121 | Health*care disparit*.mp. | 23907 |
| 122 | Health Care Disparit*.mp. | 2189 |
| 123 | Health Status Disparit*.mp. | 20112 |
| 124 | Health Disparit*.mp. | 21554 |
| 125 | Health Inequalit*.mp. | 8804 |
| 126 | Health Inequit*.mp. | 5097 |
| 127 | Medically underserved.mp. | 8778 |
| 128 | or/9-127 | 4659057 |
| 129 | systemic.mp. | 612518 |
| 130 | (anti cancer or anticancer or sact).mp. | 167643 |
| 131 | therap*.mp. | 7254862 |
| 132 | antineoplastic.mp. or Antineoplastic Agents/ | 579866 |
| 133 | cancer drug.mp. | 9717 |
| 134 | drug therapy.mp. or Drug Therapy/ | 2701520 |
| 135 | hormone therapy.mp. | 16989 |
| 136 | endocrine therapy.mp. | 10161 |
| 137 | aromatase inhibitors.mp. or Aromatase Inhibitors/ | 9099 |
| 138 | tamoxifen.mp. or Tamoxifen/ | 30382 |
| 139 | (oestrogen* or estrogen*).mp. | 220723 |
| 140 | epidermal growth factors.mp. or "EGF Family of Proteins"/ | 1234 |
| 141 | biological therapy.mp. or Biological Therapy/ | 6509 |
| 142 | antibodies monoclonal.mp. or Antibodies, Monoclonal/ | 237149 |
| 143 | Methotrexate/ | 41426 |
| 144 | immunotherapy.mp. or Immunotherapy/ | 161037 |
| 145 | 129 or 130 or 131 or 132 or 133 or 134 or 135 or 136 or 137 or 138 or 139 or 140 or 141 or 142 or 143 or 144 | 8030802 |
| 146 | Access.mp. | 438700 |
| 147 | utili?ation.mp. | 304760 |
| 148 | health care.mp. or "Delivery of Health Care"/ | 934048 |
| 149 | 146 or 147 or 148 | 1537023 |
| 150 | 8 and 128 and 145 and 149 | 443 |
| 151 | limit 150 to yr="2000 -Current" | 424 |

**Search Strategy: OVID Embase:**

| Embase <1974 to 2023 August 23> | |  |
| --- | --- | --- |
| # ▲ | Searches | Results |
| 1 | secondary.mp. | 1292604 |
| 2 | (neoplasm/ and metastasis/) or metastatic.mp. | 476517 |
| 3 | (oncology or cancer).mp. or neoplasm/ | 4666525 |
| 4 | (breast* or mammary).mp. | 995790 |
| 5 | breast cancer/ | 443065 |
| 6 | 1 or 2 | 1733152 |
| 7 | (3 and 4) or 5 | 735218 |
| 8 | 6 and 7 | 118197 |
| 9 | (sociodemographic or socioeconomic or ethnicity or race or psycho* or geograph* or location or distance or Neighbo?rhood* or rural* or inner?city or mortgage problem* or foreclosure or eviction or "housing loss" or overcrowding or Minorit* or Migration background or Racial or Racism or Ethnology or Race or Ethnic* or non?English or "Language other than" or Latino* or Latina* or Hispanic* or Whites or Caucasian* or non?white or Torres Strait Islander or Aboriginal or "Native American" or inuit or Eskimo or First Nation or Indigenous or "English as a second language" or "Foreign language" or occupations or unemployment or "Gender differences" or Gender identity or Sex Role or "Wom?n* Role?" or "M?n* Role? " or "Gender* Role?" or Servicewomen or Schooling or Educational status or Religi* or Disparit* or Inequalit* or inequit* or equity or Deprivation or Gini or Concentration index or SES or Disadvantaged or Impoverished or Poverty or Economic level or assets index or income* or Social exclusion or Social relationships or Social network* or Collective efficacy or Civil society or "Informal social control" or "Neighbo*rhood disorder" or Social Disorgani?ation or anomie or Trust or "Emotional support" or "Psychosocial support" or "Community Capital" or "Neighbo*rhood cohesion" or "Social Influence" or "Soci*context*" or "soci*-context*" or Health*care disparit* or "Health Care Disparit*" or "Health Status Disparit*" or "Health Disparit*" or "Health Inequalit*" or "Health Inequit*" or "Medically underserved").mp. | 4822638 |
| 10 | ethnic group/ or ancestry group/ or residence characteristics/ or environmental planning/ or exp marriage/ or cultural deprivation/ or cultural factor/ or cultural diversity/ or language/ or migrant/ or emigrant/ or immigrant/ or minority group/ or minority health/ or prejudice/ or racism/ or xenophobia/ or social discrimination/ or exp race relation/ or race relation/ or refugee/ or occupation/ or unemployment/ or exp gender identity/ or women's health/ or sex factor/ or exp educational status/ or educational status/ or religion/ or socioeconomics/ or social welfare/ or Poverty/ or social stigma/ or social capital/ or social control/ or social support/ or social environment/ or trust/ or social status/ or social isolation/ or social exclusion/ or anomie/ or social participation/ or health disparity/ or health care access/ or health equity/ | 1251991 |
| 11 | (housing adj2 (instability or insecurity or strain or security)).mp. | 1854 |
| 12 | (home adj2 (repossession* or ownership)).mp. | 683 |
| 13 | (repossess* adj3 (hous* or propert*)).mp. | 6 |
| 14 | (Mortgage adj2 (delinquency or arrears or debt*)).mp. | 30 |
| 15 | (living adj1 (outside or inside or near* or adjacent)).mp. | 5355 |
| 16 | (household adj2 size).mp. | 2268 |
| 17 | ((marital or marriage) adj2 status).mp. | 42646 |
| 18 | (widow* or cohabit* or divorce* or "single parent*" or "live* alone").mp. | 39355 |
| 19 | ("sex disparit*" or "sex difference?").mp. | 443989 |
| 20 | (Education* adj2 level?).mp. | 90917 |
| 21 | ((Higher or Better or Worse or Less) adj2 educated).mp. | 8761 |
| 22 | ((Higher or Better or Worse or Less) adj2 educated).mp. | 8761 |
| 23 | (Social adj2 (determinants or status or position or background or circumstance*)).mp. | 162408 |
| 24 | (socio-economic or socio-demographic).mp. | 99541 |
| 25 | (Community adj3 (cohes* or participa*)).mp. | 32323 |
| 26 | ("social" adj2 (support or participation)).mp. | 146260 |
| 27 | ("Soci*context*" or "soci*-context*").mp. | 16409 |
| 28 | 9 or 10 or 11 or 12 or 13 or 14 or 15 or 16 or 17 or 18 or 19 or 20 or 21 or 22 or 23 or 24 or 25 or 26 or 27 | 5542501 |
| 29 | systemic.mp. | 915296 |
| 30 | (anti cancer or anticancer or sact).mp. | 220580 |
| 31 | therap*.mp. | 10400432 |
| 32 | cancer drug.mp. | 13223 |
| 33 | hormone therapy.mp. | 49313 |
| 34 | endocrine therapy.mp. | 17927 |
| 35 | aromatase inhibitors.mp. | 8982 |
| 36 | tamoxifen.mp. | 77651 |
| 37 | (oestrogen* or estrogen*).mp. | 311991 |
| 38 | epidermal growth factors.mp. | 353 |
| 39 | biological therapy.mp. | 22318 |
| 40 | antibodies monoclonal.mp. | 613 |
| 41 | immunotherapy.mp. | 292822 |
| 42 | antineoplastic agent/ or drug therapy/ or aromatase inhibitor/ or tamoxifen/ or epidermal growth factor derivative/ or biological therapy/ or monoclonal antibody/ or methotrexate/ or immunotherapy/ | 1848392 |
| 43 | 29 or 30 or 31 or 32 or 33 or 34 or 35 or 36 or 37 or 38 or 39 or 40 or 41 or 42 | 11351992 |
| 44 | (Access or utili?ation or health care).mp. | 2724439 |
| 45 | health care delivery/ | 207865 |
| 46 | 44 or 45 | 2724439 |
| 47 | 8 and 28 and 43 and 46 | 1311 |
| 48 | limit 47 to yr="2000 -Current" | 1294 |

**Search Strategy: OVID PsycINFO:**

Set#: S1

Searched for: tiab(Secondary)

Databases: APA PsycInfo®

Results: 120883

Set#: S2

Searched for: (MAINSUBJECT.EXACT("Metastasis") AND MAINSUBJECT.EXACT("Neoplasms")) OR tiab(metastatic)

Databases: APA PsycInfo®

Results: 2070

Set#: S3

Searched for: tiab(Oncology OR Cancer) OR MAINSUBJECT.EXACT("Neoplasms")

Databases: APA PsycInfo®

Results: 78031

Set#: S4

Searched for: tiab(breast* or mammary)

Databases: APA PsycInfo®

Results: 21464

Set#: S5

Searched for: MAINSUBJECT.EXACT("Breast Neoplasms")

Databases: APA PsycInfo®

Results: 13165

Set#: S6

Searched for: [s1] or [s2]

Databases: APA PsycInfo®

These databases are searched for part of your query.

Results: 122833

Set#: S7

Searched for: (([s3] and [s4]) or [s5])

Databases: APA PsycInfo®

These databases are searched for part of your query.

Results: 16851

Set#: S8

Searched for: ([s6] and [s7])

Databases: APA PsycInfo®

These databases are searched for part of your query.

Results: 1194

Set#: S10

Searched for: tiab(( (housing N2 (instability or insecurity or strain or security)) OR (home N2 (repossession* or ownership)) OR (repossess* N3 (hous* or propert*)) OR (Mortgage N2 (delinquency or arrears or debt*)) OR (living N1 (outside or inside or near* or Nacent)) OR (household N2 size) OR ((marital or marriage) N2 status) OR (widow* or cohabit* or divorce* or "single parent*" or "live* alone") OR ("sex disparit*" or "sex difference?") OR (Education* N2 level?) OR ((Higher or Better or Worse or Less) N2 educated) OR (Social N2 (determinants or status or position or background or circumstance*)) OR (socio-economic or socio-demographic) OR (Community N3 (cohes* or participa*)) OR ("social" N2 (support or participation)) OR ("Soci*context*" or "soci*-context*") ) OR AB ( (housing N2 (instability or insecurity or strain or security)) OR (home N2 (repossession* or ownership)) OR (repossess* N3 (hous* or propert*)) OR (Mortgage N2 (delinquency or arrears or debt*)) OR (living N1 (outside or inside or near* or Nacent)) OR (household N2 size) OR ((marital or marriage) N2 status) OR (widow* or cohabit* or divorce* or "single parent*" or "live* alone") OR ("sex disparit*" or "sex difference?") OR (Education* N2 level?) OR ((Higher or Better or Worse or Less) N2 educated) OR (Social N2 (determinants or status or position or background or circumstance*)) OR (socio-economic or socio-demographic) OR (Community N3 (cohes* or participa*)) OR ("social" N2 (support or participation)) OR ("Soci*context*" or "soci*-context*") ) )

Databases: APA PsycInfo®

Results: 98538

Set#: S11

Searched for: (MAINSUBJECT.EXACT("Racial and Ethnic Groups") OR (MAINSUBJECT.EXACT("Systemic Racism") OR MAINSUBJECT.EXACT("Racism")) OR MAINSUBJECT.EXACT("Cultural Deprivation") OR MAINSUBJECT.EXACT("Sociocultural Factors") OR MAINSUBJECT.EXACT("Cultural Diversity") OR MAINSUBJECT.EXACT("Native Language") OR MAINSUBJECT.EXACT("Immigration") OR (MAINSUBJECT.EXACT("Minority Groups") OR MAINSUBJECT.EXACT("Sexual Minority Groups")) OR MAINSUBJECT.EXACT("Prejudice") OR MAINSUBJECT.EXACT("Marriage")) OR (MAINSUBJECT.EXACT("Social Discrimination") OR MAINSUBJECT.EXACT("Racial and Ethnic Relations") OR MAINSUBJECT.EXACT("Refugees") OR (MAINSUBJECT.EXACT("Occupational Attitudes") OR MAINSUBJECT.EXACT("Occupational Choice")) OR MAINSUBJECT.EXACT("Unemployment") OR MAINSUBJECT.EXACT("Gender Identity") OR MAINSUBJECT.EXACT("Religion") OR MAINSUBJECT.EXACT("Socioeconomic Status") OR MAINSUBJECT.EXACT("Welfare Services (Government)") OR MAINSUBJECT.EXACT("Poverty")) AND (MAINSUBJECT.EXACT("Stigma") OR MAINSUBJECT.EXACT("Social Support") OR MAINSUBJECT.EXACT("Social Status") OR MAINSUBJECT.EXACT("Social Isolation") OR MAINSUBJECT.EXACT("Social Support") OR MAINSUBJECT.EXACT("Anomie") OR MAINSUBJECT.EXACT("Social Exclusion") OR MAINSUBJECT.EXACT("Health Disparities") OR MAINSUBJECT.EXACT("Health Care Access"))

Databases: APA PsycInfo®

Results: 158140

Set#: S12

Searched for: tiab(sociodemographic or socioeconomic or ethnicity or race or psycho* or geograph* or location or distance) OR tiab(Neighbourhood* or Neighborhood rural* or inner-city or "inner city" or mortgage problem* or foreclosure or eviction or "housing loss" or overcrowding or Minorit* ) OR tiab(Migration background or Racial or Racism or Ethnology or Race or Ethnic* or non-English or "non english") OR tiab("Language other than" or Latino* or Latina* or Hispanic* or Whites or Caucasian* or non-white or "Non white" or Torres Strait Islander or Aboriginal or "Native American" or inuit or Eskimo or First Nation or Indigenous or "English as a second language") OR tiab("Foreign language" or occupations or unemployment or "Gender differences" or Gender identity or Sex Role or "Women* Role*" or "Men* Role*" or "Gender* Role*" or Servicewomen or Schooling or Educational status or Religi* or Disparit* or Inequalit* or inequit* or equity or Deprivation or Gini or Concentration index or SES or Disadvantaged or Impoverished or Poverty or "Economic level" or assets index or income* or "Social exclusion" or "Social relationships" or "Social network*" or "Collective efficacy") OR tiab(Civil society or "Informal social control" or "Neighbo*rhood disorder" or "Social Disorganisation" or "social disorganization" or anomie or Trust or "Emotional support" or "Psychosocial support" or "Community Capital" or "Neighbo*rhood cohesion" or "Social Influence" or "Soci*context*" or "soci*-context*" or "Health*care disparit*" or "Health Care Disparit*" or "Health Status Disparit*" or "Health Disparit*" or "Health Inequalit*" or "Health Inequit*" or "Medically underserved")

Databases: APA PsycInfo®

Results: 1184695

Set#: S13

Searched for: [S10] OR [S11] OR [S12]

Databases: APA PsycInfo®

These databases are searched for part of your query.

Results: 1297690

Set#: S14

Searched for: tiab(systemic or anti cancer or anticancer or sact or therap* or antineoplastic or "cancer drug" or "drug therapy" or "hormone therapy" or "endocrine therapy" or "aromatase inhibitors" or tamoxifen or oestrogen* or estrogen* or "epidermal growth factors" or "biological therapy" or "antibodies monoclonal" or Methotrexate or immunotherapy) OR MAINSUBJECT.EXACT("Antineoplastic Drugs") OR MAINSUBJECT.EXACT("Drug Therapy") OR MAINSUBJECT.EXACT("Immunotherapy")

Databases: APA PsycInfo®

Results: 528313

Set#: S15

Searched for: tiab(Access or Utilisation or utilization or "health care")

Databases: APA PsycInfo®

Results: 234225

Set#: S16

Searched for: [S8] and [S13] and [S14] and [S15]

Databases: APA PsycInfo®

These databases are searched for part of your query.

Results: 11
