# Supplementary material for: Geographic and sociodemographic access to systemic anticancer therapies for secondary breast cancer: a systematic review
Source: Syst Rev. 2024 Jan 18;13:35. doi: 10.1186/s13643-023-02382-3 (PMC10795363; doi:10.1186/s13643-023-02382-3)
Supplement: Supplementary file 3 — Additional file 3. Preferred Reporting Items for Systematic Reviews and Meta-Analyses for searching (PRISMA-S) checklist [23]. [file 13643_2023_2382_MOESM3_ESM.docx]

**Additional file 3. Preferred Reporting Items for Systematic Reviews and Meta-Analyses for searching (PRISMA-S) checklist (Rethlefsen et al, 2021):**

| **SECTION/TOPIC** | **ITEM#** | **CHECKLIST ITEM** | **Page No.** | **Line No.** |
| --- | --- | --- | --- | --- |
| **INFORMATION SOURCES AND METHODS:** | | | | |
| Database name | 1 | Name each individual database searched, stating the platform for each. | P.7 | 206 – 212 |
| Multi database searching | 2 | If databases were searched simultaneously on a single platform, state the name of the platform, listing all of the databases searched. | P.7 | 206 - 212 |
| Study registries | 3 | List any study registries searched. | N/A | N/A |
| Online resources and browsing | 4 | Describe any online or print source purposefully searched or browsed (e.g., tables of contents, print conference proceedings, web sites), and how this was done. | N/A | N/A |
| Citation searching | 5 | Indicate whether cited references or citing references were examined, and describe any methods used for locating cited/citing references (e.g., browsing reference lists, using a citation index, setting up email alerts for references citing included studies). | P.7 | 212 |
| Contacts | 6 | Indicate whether additional studies or data were sought by contacting authors, experts, manufacturers, or others. | P.9 | 253 - 255 |
| Other methods | 7 | Describe any additional information sources or search methods used. | N/A | N/A |
| **SEARCH STRATEGIES:** | | | | |
| Full search strategies | 8 | Include the search strategies for each database and information source, copied and pasted exactly as run.  **Additional file 2. Updated search strategies for Ovid CINAHL, Ovid MEDLINE, Ovid Embase and Ovid PsycINFO (August 2023).** | Additional File 2 | |
| Limits and restrictions | 9 | Specify that no limits were used, or describe any limits or restrictions applied to a search (e.g., date or time period, language, study design) and provide justification for their use. | P.8 | 224 - 225 |
| Search filters |  | Indicate whether published search filters were used (as originally designed or modified), and if so, cite the filter(s) used. | P.8 | 223 |
| Prior work | 11 | Indicate when search strategies from other literature reviews were adapted or reused for a substantive part or all of the search, citing the previous review(s). | N/A | N/A |
| Updates | 12 | Report the methods used to update the search(es) (e.g., rerunning searches, email alerts). | P.8 | 227 - 229 |
| Dates of searches | 13 | For each search strategy, provide the date when the last search occurred. | P.8 | 227 - 229 |
| **PEER REVIEW:** | | | | |
| Peer review | 14 | Describe any search peer review process. | P.8 | 228 |
| **MANAGING RECORDS:** |  |  |  |  |
| Total records | 15 | Document the total number of records identified from each database and other information sources. | P.10 | 285 - 295 |
| Deduplication | 16 | Describe the processes and any software used to deduplicate records from multiple database searches and other information sources. | P.10 | 288 - 293 |
|  |  |  |  |  |
